# A Tailored Multifunctional Anticancer Nanodelivery System for Ruthenium‐Based Photosensitizers: Tumor Microenvironment Adaption and Remodeling

**DOI:** 10.1002/advs.201901992

**Published:** 2019-11-25

**Authors:** Jin‐Hao Liang, Yue Zheng, Xiao‐Wen Wu, Cai‐Ping Tan, Liang‐Nian Ji, Zong‐Wan Mao

**Affiliations:** ^1^ MOE Key Laboratory of Bioinorganic and Synthetic Chemistry School of Chemistry Sun Yat‐Sen University Guangzhou 510275 P. R. China

**Keywords:** drug delivery, multimodal imaging, photodynamic therapy, ruthenium, tumor microenvironment

## Abstract

Ruthenium complexes are promising photosensitizers (PSs), but their clinical applications have many limitations. Here, a multifunctional nano‐platform **PDA‐Pt‐CD@RuFc** formed by platinum‐decorated and cyclodextrin (**CD**)‐modified polydopamine (**PDA**) nanoparticles (NPs) loaded with a ferrocene‐appended ruthenium complex (**RuFc**) is reported. The NPs can successfully deliver **RuFc** to the tumor sites. The release of **RuFc** from the NPs can be triggered by low pH, photothermal heating, and H_2_O_2_. The combined photodynamic and photothermal therapy (PDT‐PTT) mediated by **PDA‐Pt‐CD@RuFc** NPs can overcome the hypoxic environment of tumors from several aspects. First, the platinum NPs can catalyze H_2_O_2_ to produce O_2_. Second, vasodilation caused by photothermal heating can sustain the oxygen supplement. Third, PDT exerted by **RuFc** can also occur through the non‐oxygen‐dependent Fenton reaction. Due to the presence of **PDA**, platinum NPs, and **RuFc**, the nanosystem can be used in multimodal imaging including photothermal, photoacoustic, and computed tomography imaging. The NPs can be excited by the near‐infrared two‐photon light source. Moreover, the combined treatment can improve the tumor microenvironments to obtain an optimized combined therapeutic effect. In summary, this study presents a tumor‐microenvironment‐adaptive strategy to optimize the potential of ruthenium complexes as PSs from multiple aspects.

## Graphical abstract

A multifunctional nanocomposite for ruthenium‐based photosensitizers that can successfully adapt to and remodel tumor microenvironments is developed. The release of the photosensitizer can be triggered by low pH, photothermal heating, and H_2_O_2_. The as‐fabricated nanoparticles can accumulate in tumor tissues and show great potential for combined therapy with multimodal imaging capacities.

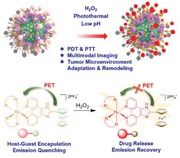

## 1 Introduction

As a widely recognized noninvasive photoactivated tumor therapy technology, photodynamic therapy (PDT) has achieved great success in clinical practice.^1^ Compared with other cancer therapies, an advantage of PDT is that it is activated by intense light exposure, resulting in controllable phototoxicity and less adverse effects.^2^ In recent years, many Ru(II) polypyridyl complexes have shown potential as effective photosensitizers (PSs) due to their visible light absorption and long‐lived triplet excited states.^3^ Upon irradiation, Ru(II) complex can photosensitize O_2_ to generate singlet oxygen (^1^O_2_) efficiently, and many Ru(II) polypyridyl complexes have good water solubility and can be well internalized by cancer cells.[qv: 3b] In addition, they can be activated by the two‐photon (TP) light source to obtain better penetration depth than visible light.^4^ Because of these properties, PSs based on Ru (II) complexes have drawn much research interest. For example, Chao et al. reported a Ru(II) polypyridyl PS with high TP absorption properties exerted its PDT effects by destroying lysosomes.[qv: 4a] Weil et al. developed a mitochondria‐targeted Ru(II) PS showed efficient growth inhibition in an acute myeloid leukemia cell line.^5^ Keyes et al. designed Ru(II) PSs modified with different localizing signal peptides, and they could cause photodamage to nuclear DNA or mitochondrial DNA.^6^ Furthermore, some new strategies were used to optimize the performance of ruthenium‐based photosensitizers, e.g., photosensitization chain‐reaction^7^ and conjugation with tumor recognition groups.^8^ A Ru(II) PS named TLD1433 is currently in the clinical stage for the treatment of nonmuscle‐invasive bladder cancer.[qv: 3a] Notably, TLD1433 was administered to bladder cancer patients in a human clinical trial since 2017, and positive clinical outcomes supporting further application of TLD1433 were achieved.[qv: 3a] However, Ru‐based PSs still have many drawbacks for further clinical applications. Ru‐based PSs lack selectivity for cancer cells/tissues, resulting in unwanted toxicity to normal cells/tissues. Most of the Ru‐based PSs reported so far function via the oxygen‐dependent PDT process relying predominantly on photosensitized generation of ^1^O_2_,^9^ while the tumor microenvironment (TME) is hypoxic.^10^

TME is recognized as a key contributor for cancer progression, metastasis, dysregulated immune responses, and drug resistance, which should be taken into consideration for the development of anticancer treatment.^11^ Malignant tumor cells produce excessive amounts of H_2_O_2_, thus the levels of H_2_O_2_ in TME are significantly increased.^12^ The upregulated glycolytic metabolism generates an acidic TME.^13^ An important feature of TME that causes fundamental limitations of PDT is the extreme hypoxia that decreases PDT efficacy by inhibiting effective ^1^O_2_ production.^14^ Moreover, the consumption of O_2_ during PDT will deteriorate tumor hypoxia.^15^ At present, several strategies are proved to be useful in overcoming tumor hypoxia during PDT process. For instance, pure O_2_ is offered to patient in a pressurized sealed chamber to promote O_2_ transport to tumors in the hyperbaric oxygen therapy.^16^ Alternatively, various O_2_‐generating/delivery materials including MnO_2_ and catalase have been used to overcome hypoxia.[qv: 14,15b,17] Another way to combat tumor hypoxia is seeking PDT independent of O_2_. For example, hydroxyl radical (•OH), the most toxic reactive oxygen species (ROS), can be generated via the Fenton reaction (Fe^2+^ + H_2_O_2_ → Fe^3+^ + OH^−^ + •OH) using H_2_O_2_.[qv: 15b,18]

**Scheme 1:**
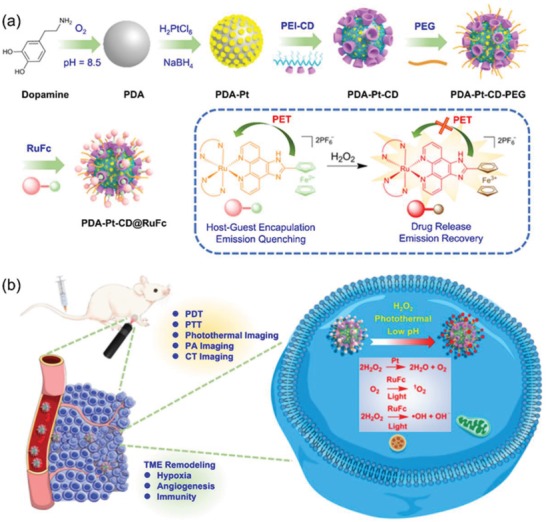
a) The construction of **PDA‐Pt‐CD@RuFc** NPs. Drug release and fluorescence recovery due to oxidation of the ferrocene group in **RuFc** by H_2_O_2_ are shown in the frame. b) Purposed action mechanisms of **PDA‐Pt‐CD@RuFc** NPs.

Although researchers are aware of the importance of optimization of nanocarriers for metallodrugs in their clinical application,^19^ tailored nanocarriers for metal‐based PSs are still rare. In this work, we designed a platinum‐polydopamine (**PDA**) hybrid nanocomposite **PDA‐Pt**. **PDA‐Pt** is modified by cyclodextrin (**CD**) groups and loaded with a Ru(II) complex (**RuFc**) through host–guest interactions to form **PDA‐Pt‐CD@RuFc** nanoparticles (NPs; **Scheme** **1**a). **PDA** can produce the photothermal effect and platinum NPs can catalyze the decomposition of H_2_O_2_ to produce O_2_. **RuFc** is appended with a ferrocene group and can be released from **CD** under stimuli including low pH, photothermal heating, and H_2_O_2_. In addition, **RuFc** can also produce •OH by photocatalytic Fenton reaction. By integrating photothermal vasodilation to enhance O_2_ supply, Pt‐catalyzed O_2_ production, and O_2_‐independent PDT process, **PDA‐Pt‐CD@RuFc** NPs can effectively resolve the hindrances faced by Ru(II)‐based PSs (Scheme 1b). Due to the presence of **PDA**, Pt, and **RuFc**, the capability of **PDA‐Pt‐CD@RuFc** NPs in multimodal (photothermal, photoacoustic (PA), and computed tomography (CT) imaging‐guided therapy was investigated. Furthermore, we also explored the improvement of TME by **PDA‐Pt‐CD@RuFc** NPs through alleviating the hypoxic pressure. In conclusion, this study provides an integrated approach to overcome the shortcomings encountered by Ru(II)‐based PSs in vivo from various respects.

## 2 Results and Discussion

### 2.1 Synthesis and Characterization

The ligand was synthesized by the literature method,^20^ and **RuFc** was obtained by refluxing the ligand and the precursor in CH_3_CH_2_OH/H_2_O (3/1, v/v) followed by anion exchange and purification by column chromatography (Scheme S1, Supporting Information). **RuFc** was characterized by electrospray ionization‐mass spectrometry (ESI‐MS; Figure S1, Supporting Information), NMR spectroscopy (Figures S2 and S3, Supporting Information), and elemental analysis. The UV/vis absorption spectra of **RuFc** in degassed CH_3_CN, CH_3_Cl_2_, and H_2_O at 298 K show intense spin‐allowed intraligand (^1^IL) absorption bands in the UV region at approximately 250–340 nm, and less intense spin‐allowed metal‐to‐ligand charge transfer (^1^MLCT) absorption bands at approximately 350–530 nm, which are typical absorption properties of Ru(II)‐polypyridyl complexes (Figure S4, Supporting Information).^21^ In CH_3_CN, CH_2_Cl_2_, and H_2_O, **RuFc** exhibits relatively weak emission with quantum yields ranging between 0.022 and 0.243 (Figure S5 and Table S2, Supporting Information).

Cyclic voltammetry measurement shows that the half‐wave potential of **RuFc** is 0.585 V (Figure S6, Supporting Information). In the presence of ROS, the fluorescence of **RuFc** is greatly enhanced (Figure S7, Supporting Information). For example, in the presence of ClO^−^ and •OH, the fluorescence intensity increases by 3.9‐ and 4.7‐fold, respectively. The phenomenon can be attributed to the oxidation of ferrocene groups, which blocks the intracellular photo‐induced electron transfer (PET) process (Scheme 1a).^22^

The synthetic procedures of **PDA‐Pt‐CD@RuFc** NPs are depicted in Scheme 1a. First, **PDA** NPs were synthesized by self‐polymerization of dopamine under alkaline conditions (pH = 8.5) with O_2_ as the oxidant according to literature methods.^23^ **PDA‐Pt** NPs are formed by in situ growth of Pt NPs on **PDA** through the reduction of H_2_PtCl_6_ by NaBH_4_ using a similar method reported in literature.^24^ **PEI‐CD** was synthesized by the substitution reaction of 6‐deoxy‐(*p*‐toluenesulfonyl)‐β‐CD (6‐OTs‐β‐CD) with the amine groups of polyethylenimine (PEI).^25^ Then, **PEI‐CD** is coated on **PDA‐Pt** NPs by noncovalent interactions to form **PDA‐Pt‐CD** NPs. In order to increase the water solubility of the NPs, methoxy poly(ethylene glycol) carboxylic acid (MPEG5000‐COOH) is coated on the surface of the NPs to form **PDA‐Pt‐CD‐PEG** NPs. Finally, **RuFc** is loaded on **PDA‐Pt‐CD‐PEG** NPs through host–guest interactions to afford **PDA‐Pt‐CD@RuFc** NPs.

**Figure 1:**
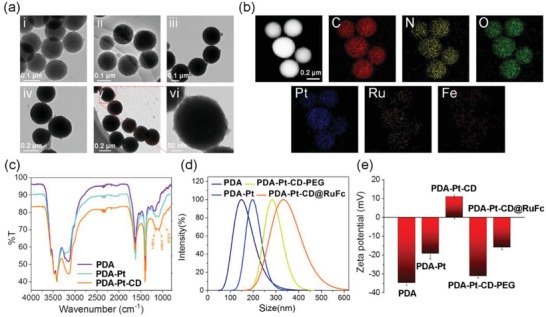
a) TEM image of: i) **PDA**, ii) **PDA‐Pt**, iii) **PDA‐Pt‐CD**, iv) **PDA‐Pt‐CD‐PEG**, and v) **PDA‐Pt‐CD@RuFc** NPs. vi) The detailed picture of (v). b) TEM elemental mapping of **PDA‐Pt‐CD@RuFc** NPs. c) The IR spectra of **PDA**, **PDA‐Pt**, and **PDA‐Pt‐CD** NPs. d) The particle size distributions of **PDA**, **PDA‐Pt**, **PDA‐Pt‐CD‐PEG**, and **PDA‐Pt‐CD@RuFc** NPs. e) The zeta‐potentials of **PDA**, **PDA‐Pt**, **PDA‐Pt‐CD**, **PDA‐Pt‐CD‐PEG**, and **PDA‐Pt‐CD@RuFc** NPs.

Transmission electron microscopy (TEM) images (**Figure** 1a) reveal that the average diameters of **PDA**, **PDA‐Pt,** and **PDA‐Pt‐CD@RuFc** NPs are about 100, 120, and 290 nm, respectively. The TEM images of **PDA‐Pt** show that the structure of **PDA** is well maintained with Pt NPs uniformly decorated on the surface of **PDA**. Meanwhile, the TEM elemental mappings (Figure 1b) show the distribution of Pt, C, N, O, Ru, and Fe elements in the same particle, which proves the formation of Pt NPs on **PDA** and the subsequent loading of **RuFc**. Quantitative energy dispersive X‐ray analysis shows the content of C, N, O, Ru, Pt, and Fe in **PDA‐Pt‐CD@RuFc** NPs (Figure S8, Supporting Information). The Fourier‐transform infrared spectra of **PDA** and **PDA‐Pt** NPs exhibit the typical peaks of the benzene ring and the hydroxy group at 1494 and 3200 cm^−1^, respectively (Figure 1c). For **PDA‐Pt‐CD** NPs, the additional peaks at 1031 and 1156 cm^−1^ are attributed to the vibrational bands of C−O and C−C bonds in **CD** moieties, respectively. The average diameters of **PDA**, **PDA‐Pt, PDA‐Pt‐CD‐PEG,** and **PDA‐Pt‐CD@RuFc** NPs determined by dynamic light scattering (DLS) measurement are about 130, 195, 280, and 350 nm, respectively (Figure 1d). It can be seen that the size distribution is relatively narrow. The hydrodynamic particle sizes of these NPs measured by DLS are larger than those obtained by TEM, as DLS gives the hydrodynamic size that corresponds to the core and the swollen corona, whereas TEM often gives the size of the dried core of NPs. The zeta potential of **PDA** is −34.73 ± 1.25 mV, and it becomes −19.22 ± 2.87 mV after modification of the Pt NPs (Figure 1e). The result is in accordance with the literature report.^24^ The zeta potential of **PDA‐Pt‐CD** becomes 11.09 ± 0.82 mV after coated with the positively charged **PEI‐CD**, and then turns negative again after the absorption of the negatively charged PEG. As expected, the loading **RuFc** with positively charges results in smaller negative potential values of **PDA‐Pt‐CD@RuFc** NPs. After an incubation for 48 h, **PDA‐Pt‐CD@RuFc** NPs are still dispersed uniformly in phosphate buffered saline (PBS), cell culture medium, and fetal bovine serum (FBS) with no precipitate observed (Figure S9a, Supporting Information). The UV/vis absorption spectra of **PDA‐Pt‐CD@RuFc** NPs in PBS remain almost unchanged after 2 days (Figure S9b, Supporting Information). The results show that **PDA‐Pt‐CD@RuFc** NPs are stable in these media and suitable for biological applications.

### 2.2 Drug Loading and Releasing Properties

**Figure 2:**
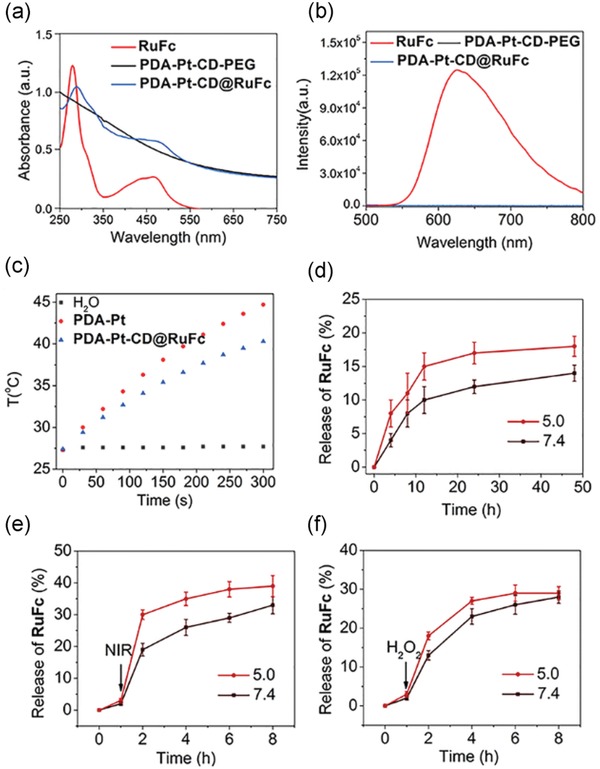
a) UV/vis spectra and b) fluorescence emission of **RuFc** (10 × 10^−6^ m), **PDA‐Pt‐CD‐PEG** (30 µg mL^−1^), and **PDA‐Pt‐CD@RuFc** (30 µg mL^−1^) NPs. c) The temperature changes of the **PDA‐Pt** (100 µg mL^−1^) and **PDA‐Pt‐CD@RuFc** (100 µg mL^−1^) upon irradiation at 808 nm (1 W cm^−2^) for different periods of time. d–f) In vitro pH‐dependent (d), photothermal‐triggered (e), and H_2_O_2_‐responsive (f) release of **RuFc** from **PDA‐Pt‐CD@RuFc** NPs at pH 5.0 and 7.4. The samples were irradiated with an 808 nm laser irradiation (1 W cm^−2^) or mixed with 100 µL H_2_O_2_ (100 × 10^−3^ m) at 1 h.

Both **PDA** and **PDA‐Pt‐CD‐PEG** NPs show strong absorbance in the near‐infrared (NIR, wavelength = 700–1100 nm) region (**Figure** 2a). After loading with **RuFc**, another two peaks at approximately 275 and 470 nm are detected for **PDA‐Pt‐CD@RuFc** NPs. According to the law of Lambert–Beer, the loading rate of **RuFc** is calculated to be 8.5%. Accordingly, the loading of **RuFc** onto **PDA‐Pt‐CD‐PEG** leads to full quenching of the emission (Figure 2b), which can be attributed to the quenching effect of **PDA** and Pt NPs on chromophores.

**PDA‐Pt** NPs show effective photothermal conversion capacities, and the photothermal performance is maintained after **CD** modification and **RuFc** loading (Figure 2c and Figure S9, Supporting Information). **PDA‐Pt‐CD@RuFc** NPs show a concentration‐dependent photothermal conversion efficacy. The efficiency of photothermal conversion of **PDA‐Pt‐CD@RuFc** NPs is calculated to be 44.5%, which is in the same range of those reported for other **PDA**‐based NPs.[qv: 23b,c] The photothermal conversion efficiency of **PDA‐Pt‐CD@RuFc** NPs is also higher than those of the commercial gold nanoshells (13.0%) and gold nanorods (21%).^26^

Responsive drug release prevents the immature release of the drug before it reaches the tumor tissue, which is beneficial for targeted cancer therapy. We find that three external stimuli (low pH, H_2_O_2_, and photothermal heating) can enhance the release of **RuFc** from **PDA‐Pt‐CD@RuFc** NPs (Figure 2d–f). After 48 h incubation, the release ratios of the **RuFc** are 18 ± 1.5% and 14 ± 1.2% at pH 5.0 and pH 7.4, respectively. The TME tends to be acidic (pH 5.7–7.8),^27^ and the environment in lysosomal (pH 4.5–5.5)/endosome (pH from 6.8 to <5.5) is also acidic.^28^ The release of **RuFc** will be facilitated under these conditions. Upon irradiation at 808 nm, the release ratios of **RuFc** at pH 5.0 and pH 7.4 reach 39 ± 3.3% and 33 ± 2.7%, respectively, which may be caused by the weakened interactions between **RuFc** and the NPs. In the presence of H_2_O_2_, the release rates of **RuFc** are 29 ± 1.7% and 28 ± 1.6% at pH 5.0 and pH 7.4, respectively. In the presence of the oxidants, **RuFc** can be released from the **CD**s due to the oxidation of the ferrocene groups.^29^ Both acidity and excessive H_2_O_2_ are important characteristics of TME, which are favorable for the release of **RuFc**. In addition, photothermal conditions can be applied externally to stimulate the release of **RuFc**. Further, the intracellular drug release responsive to different stimuli was investigated. Both lasers (450 and 808 nm) and H_2_O_2_ can stimulate the release of **RuFc** from **PDA‐Pt‐CD@RuFc** NPs, as indicated by the recovery of the fluorescence (Figure S11, Supporting Information). The phenomenon is more obvious as the cellular acidity increases (Figure S12, Supporting Information). The cellular distribution of **RuFc**, **PDA‐Pt‐CD‐PEG,** and **PDA‐Pt‐CD@RuFc** was measured by inductively coupled plasma‐mass spectrometry (ICP‐MS). After incubated in 4T1 cells for 12 h, **RuFc** tends to localize in mitochondria (Figures S13 and S14, Supporting Information). However, **PDA‐Pt‐CD‐PEG** and **PDA‐Pt‐CD@RuFc** are mainly detected in the cytoplasm.

### 2.3 Catalyze the Deposition of H_2_O_2_

**Figure 3:**
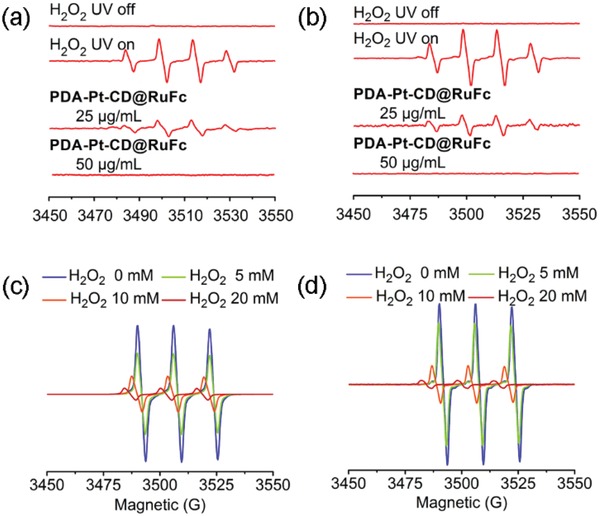
a,b) Effect of **PDA‐Pt‐CD@RuFc** NPs on •OH production in the H_2_O_2_/UV system at pH 6.5 (a) and pH 7.4 (b). c,d) Detection of the O_2_ (EPR spin label oximetry) produced by reaction catalyzed by **PDA‐Pt‐CD@RuFc** NPs (50 µg mL^−1^) at pH 6.5 (c) and pH 7.4 (d).

As pH can influence the catalytic capacity of nanozymes,^30^ the catalase‐like activity of **PDA‐Pt‐CD@RuFc** NPs is measured at pH 7.4 and 6.5. UV light can decompose H_2_O_2_ to produce •OH, and the degree of H_2_O_2_ decomposition can be determined by quantifying the content of •OH using the radical trap 5,5‐dimethyl‐1‐proline‐*N*‐oxide (DMPO). The four characteristic peaks of DMPO/•OH adducts in the electron spin resonance spectra are weakened in the presence of **PDA‐Pt‐CD@RuFc** (**Figure** **3**a,b) and the catalyst (Figure S15, Supporting Information). The reaction between **PDA‐Pt‐CD@RuFc** NPs and H_2_O_2_ under different conditions were also investigated be measuring the absorbance at 240 nm of H_2_O_2_. The concentration of H_2_O_2_ is decreased as the reaction proceeds in the sample containing **PDA‐Pt‐CD@RuFc** NPs (Figure S16, Supporting Information).

**PDA‐Pt‐CD@RuFc** NPs can decompose H_2_O_2_ to generate O_2_ like the catalase, as evidenced by the gas bubbles in the tubes and H_2_O_2_ at both pH 7.4 and 6.5 (Figure S17, Supporting Information). The O_2_ produced by the catalytic reaction is also detected by the electron paramagnetic resonance (EPR) measurement using the O_2_‐sensitive spin‐label probe 3‐carbamoyl‐2,2,5,5‐tetramethyl‐3‐pyrroline‐l‐yloxyl (CTPO). As the concentration of H_2_O_2_ increases, the signal of CTPO decreases gradually in the presence of **PDA‐Pt‐CD@RuFc** NPs (Figure 3c,d), which is similar to that observed for the catalyst (Figure S14, Supporting Information). In all these experiments, **PDA** does not show a catalytic activity for H_2_O_2_ decomposition, which confirms that the catalase‐like properties originate from the Pt NPs.

### 2.4 Catalyze the Photo‐Fenton Reaction and Photosensitize the Generation of ^1^O_2_

**Figure 4:**
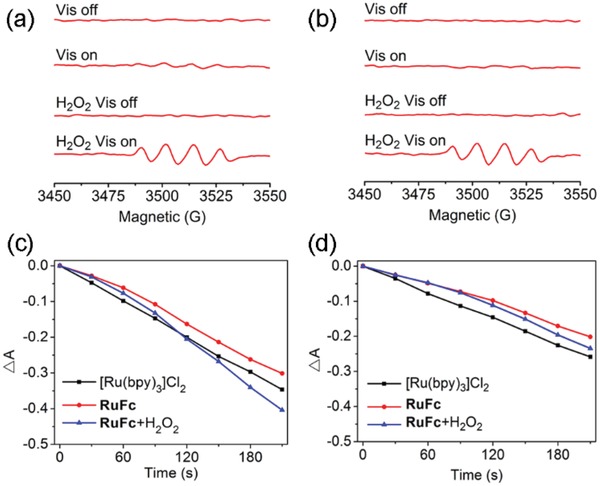
a,b) Production of •OH through photo‐Fenton reaction catalyzed by **RuFc** (50 × 10^−6^ m) at pH 6.5 (a) and pH 7.4 (b). Irradiation conditions: 450 nm, 20 mW cm^−2^, 3 min. c,d) Determination of the ^1^O_2_ by **RuFc** using ABDA (100 × 10^−6^ m) as the probe under visible light irradiation in the absence or presence of H_2_O_2_ (100 × 10^−6^ m) at pH 6.5 (c) and pH 7.4 (d). The solutions were irradiated with a 450 nm laser (20 W cm^−2^) for different time periods.

Next, we studied the reaction of **RuFc** with H_2_O_2_ to produce •OH through photo‐Fenton reaction using DMPO as the radical trap. **RuFc** can effectively generate •OH in the presence of H_2_O_2_ upon visible light illumination at both pH 6.5 and 7.4 (**Figure** 4a,b). Upon visible light irradiation, the absorbance of 9,10‐anthracenediyl‐bis(methylene)dimalonic acid (ABDA, an ^1^O_2_ indicator) decreases quickly (Figure 4c,d), which indicates that **RuFc** can efficiently photosensitize the generation of ^1^O_2_. The ^1^O_2_ quantum yields of **RuFc** are measured to be 0.098 and 0.152 at pH 7.4 and 6.5, respectively (Table S3, Supporting Information). In the presence of H_2_O_2_ (100 × 10^−6^ m, a concentration relevant to the tumor environment), the yields of ^1^O_2_ are increased at both pH 6.5 and 7.4 (Table S3, Supporting Information). The phenomena may be attributed to the oxidation of ferrocene groups by H_2_O_2_, which restores the fluorescence (Figure S7, Supporting Information) and photosensitizing properties by blocking the PET process.

### 2.5 In Vitro Combined PDT‐PTT (Photothermal Therapy) Activities

**Figure 5:**
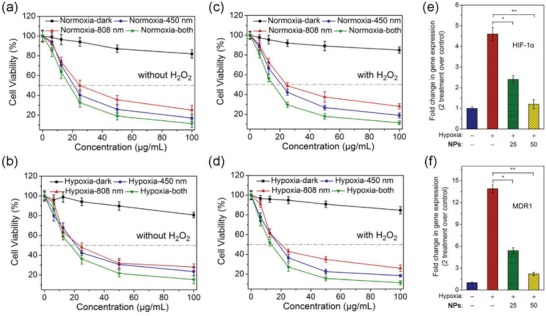
In vitro combined PDT‐PTT activities of **PDA‐Pt‐CD@RuFc** NPs measured on 4T1 cells. Cells were cultured under hypoxia (1% O_2_) or normoxia (21% O_2_) in the absence or presence of H_2_O_2_. a) Normoxia; H_2_O_2_ (0 × 10^−6^ m). b) Hypoxia; H_2_O_2_ (0 × 10^−6^ m). c) Normoxia; H_2_O_2_ (3 × 10^−3^ m). d) Hypoxia, H_2_O_2_ (3 × 10^−3^ m). Irradiation conditions: 450 nm, 17 mW cm^−2^, 1 min; 808 nm, 1 W cm^−2^, 10 min. e,f) The expression of HIF‐1α (e) and MDR1 (f) genes in 4T1 cells treated with **PDA‐Pt‐CD@RuFc** (25 or 50 µg mL^−1^) under hypoxia (1%) or normoxia (21%). Incubation time: 6 h. Statistical *p*‐value: *p < 0.05, **p < 0.01.

Next, we studied the combined PDT‐PTT effects of **PDA‐Pt‐CD@RuFc** NPs on 4T1 breast cancer cells in vitro. **PDA‐Pt‐CD@RuFc** NPs shows low toxicity in the absence of light under both normoxia and hypoxia (**Figure** 5a–d). Appreciable cytotoxicities are observed for **PDA‐Pt‐CD@RuFc** NPs upon either photothermal (808 nm) or photodynamic (450 nm) treatment, and a good synergetic effect can be obtained for PDT‐PTT combined treatment. Under normoxia, the PDT effect is elevated in the presence of H_2_O_2_ because it can stimulate the release of **RuFc** and enhance the photon‐Fenton reaction (Figure 5a,c). In the presence of H_2_O_2_ under hypoxia, the efficacy of **PDA‐Pt‐CD@RuFc** NPs with combined **PDT‐PTT** treatment is similar to that obtained under normal conditions (Figure 5a,d). Moreover, **PDA‐Pt‐CD@RuFc** treatment can reduce the expression of hypoxia‐inducible factor 1a (HIF‐1a) and multi‐drug resistance (MDR1) genes as determined by real‐time polymerase chain reaction (Figure 5e,f). The expression of these genes are known to be induced by hypoxia.^31^ The results indicate that **PDA‐Pt‐CD@RuFc** NPs can well adapt to and improve the TME to produce a synergistic PDT‐PTT therapeutic effect.

The in vitro combined PDT‐PTT activities were also tested on human breast cancer MB‐MDA‐231 cells, human cervical carcinoma HeLa cells, and human normal hepatic LO2 cells (Figure S19, Supporting Information). **PDA‐Pt‐CD@RuFc** NPs show very good combined therapeutic activity for cancer cells, especially for MB‐MDA‐231 cells. For normal cells, the inhibitory activity of the NPs is lower than that observed for tumor cells.

Since the penetration of visible light is limited, we also attempt to evaluate the two‐photon PDT (TPPDT) efficacy of **PDA‐Pt‐CD@RuFc** NPs in both 2D cells and multicellular tumor spheroids (MCTSs; Figure S20a, Supporting Information). The 3D MCTSs model can simulate the hypoxic TME and reflect the penetration capability of TP light source. First, the impact of TPPDT on viability of 2D 4T1 cells was visualized by Calcein AM staining. The viability of cells with photothermal (808 nm) or TP photodynamic (810 nm) treatment decreases significantly in both 2D and 3D models. After the combined TPPDT‐PTT therapy, the fluorescence of Calcein is further reduced. Cell viability assay also confirms the low toxicity of **PDA‐Pt‐CD@RuFc** toward MCTSs in the dark and high toxicity upon TPPDT‐PTT treatment (Figure S20b, Supporting Information). The results show that the PDT effects of **PDA‐Pt‐CD@RuFc** NPs can also be excited by the TP light source with higher penetration depth.

### 2.6 In Vitro Anticancer Mechanism

**Figure 6:**
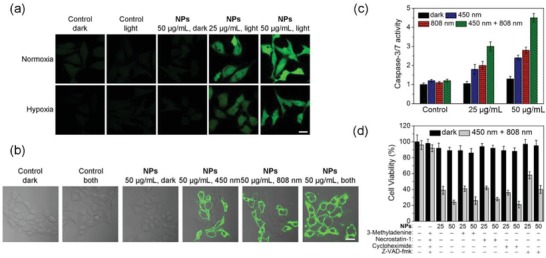
a) Intracellular ROS levels detected by H_2_DCFDA staining in 4T1 cells treated with **PDA‐Pt‐CD@RuFc** NPs in combination with light irradiation. Cells were cultured under hypoxia (1% O_2_) or normoxia (21% O_2_) atmosphere and treated with the NPs. Irradiation conditions: 450 nm, 17 mW cm^−2^, 1 min. b) Detection of apoptosis by Annexin V staining in 4T1 cells with PDT‐PTT combined treatment mediated by **PDA‐Pt‐CD@RuFc**. c) Detection of caspase‐3/7 activity in 4T1 cells with PDT‐PTT combined treatment mediated by **PDA‐Pt‐CD@RuFc**. d) The impact of different inhibitors on the viability of 4T1 cells with PDT‐PTT combined treatment mediated by **PDA‐Pt‐CD@RuFc**. Irradiation conditions for (b), (c), and (d): 450 nm, 17 mW cm^−2^, 1 min; 808 nm, 1 W cm^−2^, 10 min.

As PDT acted through elevation of ROS, we first detected the cellular ROS levels in cells using 2′,7′‐dichlorodihydrofluorescein diacetate (H_2_DCFDA) staining upon treatment.^32^ The level of ROS in the cells treated with **PDA‐Pt‐CD@RuFc** NPs in combination with light increases significantly under normoxia (**Figure** 6a). The capability of **PDA‐Pt‐CD@RuFc** NPs to photosensitize the generation of ROS under hypoxia is not obviously diminished, indicating that **PDA‐Pt‐CD@RuFc**‐mediated PDT can overcome tumor hypoxia.

Subsequently, we studied the effects of ROS on the integrity of cellular organelles. First, we investigated the lysosomal damage in **PDA‐Pt‐CD@RuFc**‐treated 4T1 cells by Magic Red MR‐(RR)_2_ staining. The control cells show dot‐like red fluorescence mostly localized in the lysosomes. In contrast, **PDA‐Pt‐CD@RuFc**‐treated cells with light irradiation show diffused red fluorescence (Figure S21, Supporting Information). The changes in mitochondrial membrane potential (MMP) was evaluated by 5,5′,6,6′‐tetrachloro‐1,1′‐3,3′‐tetraethyl‐benzimidazolylcarbocyanine iodide (JC‐1) staining.^33^ Upon irradiation, a marked decrease in MMP, indicated by the decrease in JC‐1 red/green fluorescence ratio, can be observed in **PDA‐Pt‐CD@RuFc**‐treated cells (Figure S22, Supporting Information). The collapse of MMP is more pronounced in cells subjected to **PDA‐Pt‐CD@RuFc**‐mediated combined PDT‐PTT therapy. Accordingly, **PDA‐Pt‐CD@RuFc** NPs cause a significant decrease in adenosine triphosphate production in the presence of light (Figure S23, Supporting Information).

Next, we used the Annexin V staining to detect the externalization of phosphatidylserine, a key event during early apoptosis. After 4T1 cells are incubated with **PDA‐Pt‐CD@RuFc** NPs and exposed to light, the proportion of Annexin V‐positive cells increases significantly, as measured by confocal microscopy (Figure 6b). The phenomenon is more obvious for cells with combined PDT‐PTT treatment. Caspase 3/7 activity assay also confirms that **PDA‐Pt‐CD@RuFc** NPs induce cell death through the apoptotic pathway (Figure 6c). Using different inhibitors, we validated the cell‐death modes by which **PDA‐Pt‐CD@RuFc** NPs kill the cancer cells through combined therapy. In the presence of the autophagy inhibitor (3‐methyladenine),^34^ the necrosis inhibitor (necrostatin‐1, inhibitor of RIP1 kinase),^35^ and the paraptosis inhibitor (cycloheximide, an inhibitor of protein and RNA biosynthesis),^36^ no significant changes in cell variability are detected (Figure 6d). However, in the presence of the apoptosis inhibitor (Z‐VAD‐fmk, a pan‐caspase inhibitor),^37^ the cell viability increases obviously. These experiments show that the combined PDT‐PTT therapy kills tumor cells mainly by inducing apoptosis.

### 2.7 In Vivo Imaging, Biodistribution, Metabolism, and Anticancer Properties

**Figure 7:**
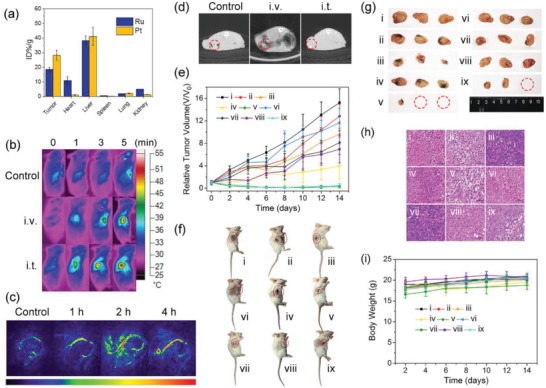
a) Biodistribution of Ru and Pt elements in different organs 24 h after i.v. injection of **PDA‐Pt‐CD@RuFc** NPs. The values are presented as the percentage of injected dose per g of the collected organs based on three mice per group. b) Thermal images of 4T1‐tumor‐bearing mice treated with **PDA‐Pt‐CD@RuFc** NPs (200 µL, 1 mg mL^−1^, 4 h) and exposed to an 808 nm laser (1 W cm^−2^) for 0, 1, 3, and 5 min. c) In vivo PA imaging of 4T1‐tumor‐bearing mice i.v. injected with **PDA‐Pt‐CD@RuFc** NPs (200 µL, 1 mg mL^−1^) for different time intervals. d) In vivo CT imaging of 4T1‐tumor‐bearing mice treated with **PDA‐Pt‐CD@RuFc** NPs (100 µL, 5 mg mL^−1^). The images were taken 30 min after i.t. injection and 2 h after i.v. injection. e) Tumor growth curves of different groups of mice (5 mice per group). f) Representative photos of different groups of mice after various treatments were taken at day 14. The tumor sites were marked with red dashed circles. g) Representative photos of tumors collected from different groups of mice at the end of treatment. The red dashed circles represent tumors that completely disappear. h) H&E staining of 4T1 tumor tissues of different groups of mice. i) Body weight curves of different groups of mice. The mice in (e)–(i) are divided into nine groups: i) control; ii) dark, i.t.; iii) 450 nm, i.t.; iv) 808 nm, i.t.; v) 808 nm + 450 nm, i.t.; vi) dark, i.v.; vii) 450 nm, i.v.; viii) 808 nm, i.v.; ix) 808 nm + 450 nm, i.v. Irradiation conditions: 450 nm, 12 W cm^−2^, 5 min; 808 nm, 1 W cm^−2^, 3 min.

Next, we studied the in vivo organ distribution and metabolism of **PDA‐Pt‐CD@RuFc** NPs. ICP‐MS measurements of Ru and Pt show that both elements are enriched in tumor and liver tissues 24 h post intravenous (i.v.) injection (**Figure** 7a), which can be attributed to the enhanced permeability and retention effects of nanomaterials. In addition, we find that a small amount of Ru accumulates in heart and kidneys, which may be due to the fact that the released small molecules can circulate between organs more easily. After 7 days, the contents of Ru and Pt in the organs are greatly reduced (Figure S24, Supporting Information), which indicates that **PDA‐Pt‐CD@RuFc** NPs can be metabolized in vivo.

Considering the potent photothermal effect of **PDA‐Pt‐CD@RuFc**, we studied the capability of it as a photothermal imaging agent. It can be seen that after 4 h treatment and illumination at 808 nm for 5 min, the temperature of the tumor area increases to 53 °C for intratumoral (i.t.) injection (Figure 7b). For mice with i.v. injection, the temperature of the tumor area gradually increases to 49 °C in 4 h, which shows that **PDA‐Pt‐CD@RuFc** NPs have good tumor‐targeting capabilities. Accordingly, after the mice were i.v. injected with the **PDA‐Pt‐CD@RuFc** NPs, obvious PA signals can be detected in the tumor site (Figure 7c). Moreover, because of the presence of heavy metal elements (Pt and Ru), **PDA‐Pt‐CD@RuFc** NPs can also be applied in CT imaging (Figure 7d). After i.t. or i.v. injection of **PDA‐Pt‐CD@RuFc** NPs, increased CT signals can be observed in the tumor sites (Figure 7d). Our results show that **PDA‐Pt‐CD@RuFc** NPs can be successfully applied in multimodal tumor imaging, which provides possibilities to integrate the functions of diagnosis and treatment together.

The in vivo antitumor potency of **PDA‐Pt‐CD@RuFc** NPs was then evaluated in mice bearing 4T1 tumors by i.t./i.v. injection. For both injection methods, PDT or PTT along shows a certain tumor inhibition efficiency (Figure 7e–g). Notably, the tumors almost completely disappear for PDT‐PTT combined therapy after treatment for 14 days. The in vivo anticancer efficacy was further evaluated by analyzing the tumor histological sections using hematoxylin and eosin (H&E) staining at the end of the treatment. Among these samples, the most serious cell death is observed for mice with **PDA‐Pt‐CD@RuFc‐**mediated combined therapy (Figure 7h). The results show that the multiple adaptive strategies of **PDA‐Pt‐CD@RuFc** NPs is effective in tumor treatment.

In order to assess the biocompatibility of **PDA‐Pt‐CD@RuFc** NPs in vivo, the body weight and H&E staining on histological sections of major organs were analyzed. The body weight of mice is not significantly reduced for all the groups (Figure 7i). No obvious pathological changes are present on the main tissues 14 days after the i.v. injection of **PDA‐Pt‐CD@RuFc** NPs (Figure S25, Supporting Information). The results indicate that the **PDA‐Pt‐CD@RuFc** NPs have low systemic toxicity.

### 2.8 Modulation of TME

**Figure 8:**
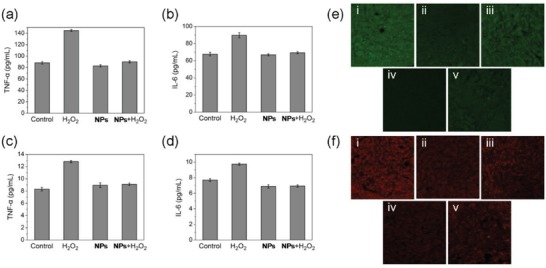
a) TNF‐a and b) IL‐6 level in 4T1 cells after various treatments. Raw 264.7 cells were treated with the supernate of 4T1 cells exposed to different treatments. c) TNF‐a and d) IL‐6 level in sera of mice after various treatments. e,f) Immunofluorescence images of HIF‐1α (e) and VEGF (f) of tumors after treatment with **PDA‐Pt‐CD@RuFc**. The mice are divided into five groups: i) control; ii) dark; iii) 450 nm; iv) 808 nm; v) 808 nm + 450 nm. Irradiation conditions: 450 nm, 12 W cm^−2^, 5 min; 808 nm, 1 W cm^−2^, 3 min.

As tumor hypoxia is closely related to many events such as inflammation, angiogenesis, and metastasis,^38^ we examined the effects of **PDA‐Pt‐CD@RuFc** on remodeling of TME. Both at the cellular level (**Figure** 8a,b) and in tumor slices (Figure 8c,d), the treatment of **PDA‐Pt‐CD@RuFc** NPs can significantly down‐regulate the expression of tumor necrosis factor (TNF)‐ɑ and interleukin 6 (IL‐6) that are markers of inflammatory TME. The expression of two important genes related to hypoxia, namely, HIF‐1ɑ and vascular endothelial growth factor (VEGF), was also investigated by immunofluorescence (Figure 8e,f) and immunohistochemical assays (Figure S26, Supporting Information). HIFs are key proteins regulating cellular response to hypoxia, and they can be activated by hypoxic TME.^39^ VEGF is a key regulator of many cancerous events including angiogenesis and metastasis.^40^ After combined treatment mediated by **PDA‐Pt‐CD@RuFc** NPs, both the expression of HIF‐1ɑ and VEGF are decreased significantly. Moreover, a decrease in the expression of cluster of differentiation‐31 (CD31, an endothelial cell marker gene)^41^ and an increase in the expression of ɑ‐smooth muscle actin (ɑ‐SMA, a marker of mature vascular endothelial cells)^42^ are also detected. The results show that **PDA‐Pt‐CD@RuFc** NPs can improve the hypoxic TME, which may further enhance their therapeutic effect.

## 3 Conclusions

The potential of ruthenium‐based PSs is limited by lacking of tumor‐targeting capability and the decline of PDT efficacy in TME, especially hypoxia. In this work, we report a multifunctional nano drug delivery system **PDA‐Pt‐CD@RuFc** that is modified with **CD** and loaded with a Ru(II) complex through host–guest interactions. The release of **RuFc** can be triggered by low pH, photothermal heating, and H_2_O_2_. The **PDA‐Pt‐CD@RuFc** NPs can accumulate in tumor tissues and show great potential for combined PDT‐PTT therapy with multimodal imaging capacities, including photothermal, PA, and CT. We prove that the nanosystem can overcome the hypoxic TME in many respects: 1) Pt NPs can catalyze the decomposition of H_2_O_2_ to produce O_2_; 2) photothermal heating can induce vasodilation; 3) **RuFc** can produce •OH through the oxygen‐independent photo‐Fenton reaction. Moreover, **PDA‐Pt‐CD@RuFc** NPs can remodel the TME in several aspects including hypoxia, immunity, and angiogenesis. In summary, the work offers multiple adaptive strategies to optimize the in vivo potential of ruthenium‐based PSs.

## 4 Experimental Section

*Materials*: RuCl_3_ ⋅ *n*H_2_O, 4,7‐diphenyl‐1,10‐phenanthroline, cisplatin, and chloroplatinic acid (H_2_PtCl_6_) were purchased from J&K Chemical, China. Dimethyl sulfoxide (DMSO), 3‐(4,5‐dimethyl‐2‐thiazolyl)‐2,5‐diphenyl‐2*H*‐tetrazolium bromide, Dulbecco's modified Eagle's medium, FBS, and the antibiotics (penicillin/streptomycin) were purchased from Gibco BRL. H_2_DCFDA, Annexin V‐FITC assay kit and JC‐1 were obtained from Sigma‐Aldrich (USA). Calcein AM were purchased from Shanghai Yusheng Biotechnology Co. Ltd. (China). Magic Red MR‐(RR)_2_ was purchased from Immunochemistry Tech (USA). The primary and secondary antibodies were obtained from Cell Signaling Technology (USA). All the other chemicals were of analytical grade obtained through commercial resources. Deionized water was purified by a Milli‐Q water purification system (Millipore, USA).

*General Instruments*: ESI‐MS was carried out on an LTQ XL H linear ion trap mass spectrometer. ^1^H NMR was carried out on a Bruker Avance III 400 MHz spectrometer (Germany). Shifts were referenced relative to the internal solvent signals. The element analysis (C, H, N) was determined by an Elemental Vario EL CHNS analyzer (Germany). Flow cytometry was carried by a BD FACSCalibur flow cytometer (Becton Dickinson, Oxford, UK). Confocal microscopic observations were taken on confocal microscopy (LSM 710, Carl Zeiss, Göttingen, Germany). The electrochemical measurement was performed on an electrochemical workstation (CHI760E).

*Synthesis of [Ru(DIP)_2_(FIP)](PF_6_)_2_* (***RuFc***): The synthetic route of **RuFc** is depicted in Scheme S1 in the Supporting Information. *Cis*‐[Ru(DIP)_2_Cl_2_] · 2H_2_O^43^ and **FIP** (2‐ferrocenyl‐1*H*‐imidazo[4,5‐f][1,10]‐phenanthroline)^44^ were synthesized by literature methods. The precursor *cis*‐[Ru(DIP)_2_Cl_2_] · 2H_2_O (200 mg, 0.239 mmol) and the ligand **FIP** (97 mg, 0.239 mmol) were dissolved in 40 mL ethanol/water (75:25, v/v). The mixture was refluxed for 6 h under the protection of a nitrogen atmosphere. The solvent was removed by rotary vacuum evaporation. A small amount of acetonitrile was used to dissolve the crude product. The product was purified on a neutral alumina column using acetonitrile as the eluent for chromatographic separation. Concentrated aqueous solution of NH_4_PF_6_ was used to precipitate the product. After several times of washing with water and ether, the desired complex was dried in vacuum. Complex **RuFc** was obtained as deep red powder. Yield: 280.8 mg (84%). ^1^H NMR (400 MHz, DMSO‐*d*_6_) δ 13.86 (s, 1H), 9.09 (d, *J* = 8.3, 2H), 8.35 (d, *J* = 5.5, 2H), 8.32–8.20 (m, 6H), 8.16 (d, *J* = 5.3, 2H), 7.92 (dd, *J* = 8.4, 5.3, 1H), 7.89–7.85 (m, 1H), 7.82 (d, *J* = 5.5, 2H), 7.76 (t, *J* = 5.7, 2H), 7.74–7.57 (m, 20H), 7.23 (d, *J* = 7.5, 1H), 7.16 (d, *J* = 7.7, 1H), 5.21 (t, *J* = 1.9, 2H), 4.61 (t, *J* = 1.9, 2H), 4.19 (s, 5H). ^13^C NMR (126 MHz, DMSO) δ 155.46, 152.99, 152.75, 150.48, 148.51, 148.36, 148.23, 135.96, 135.92, 130.42, 130.35, 130.10, 129.61, 128.60, 128.53, 127.00, 126.50, 74.21, 70.52, 70.07, 68.52. ESI‐MS (CH_3_CN): *m*/*z* 585.45 [M−2PF_6_]^2+^. Elemental analysis: calcd (%) for C_71_H_48_F_12_FeN_8_P_2_Ru⋅H_2_O: C, 57.70; H, 3.41; N, 7.58; found: C, 57.43; H, 3.35; N, 7.55.

*Synthesis of* ***PDA‐Pt***: The **PDA** NPs were prepared following a literature procedure.^23^ **PDA‐Pt** was formed by in situ growth of Pt NPs on PDA through the reduction of H_2_PtCl_6_ by NaBH_4_ using a similar method reported in literature.^24^

*Synthesis of* ***PEI‐CD***: **PEI‐CD** was synthesized by the substitution reaction of 6‐OTs‐β‐CD with the amine groups of PEI following the literature method.^25^

*Synthesis of* ***PDA‐Pt‐CD***: **PDA‐Pt** and **PEI‐CD** were dispersed in 10 × 10^−3^ m Tris‐HCl buffer solution (10 × 10^−3^ m, pH = 8.5) at a 10:1 mass ratio. The mixture was stirred at room temperature for 20 h, centrifuged, and washed with a large amount of deionized water. The product **PDA‐Pt‐CD** was redispersed in ultra‐pure water for the next step.

*Synthesis of* ***PDA‐Pt‐CD‐PEG***: MPEG5000‐COOH (5 mg, 1 eq) was dissolved in DMSO (0.5 mL). 1‐[bis(dimethylamino)methylene]‐1*H*‐1,2,3‐triazolo[4,5‐b]pyridinium 3‐oxid hexafluorophosphate (1.2 eq) and diisopropylethylamine (2 eq) were added and the mixture was stirred at room temperature for 30 min. After the addition of **PDA‐Pt‐CD** (1.5 mg mL^−1^ in water, 10 mL), the mixture was vigorously stirred overnight at room temperature, centrifuged, and washed with a large amount of deionized water. The solid obtained was redispersed in ultrapure water for the next step.

*Synthesis of* ***PDA‐Pt‐CD@RuFc***: **PDA‐Pt@CD‐PEG** (10 mg, 1 mg mL^−1^ in water) and **RuFc** (500 µL, 1.2 mg in water) were mixed by ultrasound for 10 min. The mixture was stirred at room temperature for 24 h to make sure that **RuFc** was fully loaded onto the NPs. After centrifugation, free **RuFc** was removed by repeated washing with ultrapure water. **PDA‐Pt‐CD@RuFc** was redispersed in PBS (pH 7.4) and stored at 4 °C.

*Catalase‐Like Activity of NPs*: The decomposition of H_2_O_2_ under UV light could produce the short‐lived •OH that could be captured by DMPO to form a relatively stable adduct DMPO/•OH. The EPR signals of DMPO/•OH adducts were characterized by a 1:2:2:1 quadruple peak. The catalase‐like activity of **PDA‐Pt‐CD** and **PDA‐Pt‐CD@RuFc** NPs was investigated by EPR using the reaction. Briefly, DMPO (50 × 10^−3^ m) and H_2_O_2_ (30 × 10^−3^ m) were incubated with the NPs (25 and 50 µg mL^−1^) or the catalase (1 and 2 U mL^−1^) for 3 min in buffered solutions at different pH (6.5 and 7.4). The EPR spectra were measured on a Bruker A300 X‐band EPR spectrometer (modulation amplitude, 1 G; sweep width, 100 G; microwave power, 20 mW; time constant, 163.84 ms).

*Decomposition of H_2_O_2_ by NPs*: The amount of H_2_O_2_ remaining after the reaction were quantified by its absorption at 240 nm. Briefly, the decomposition experiment was carried out by adding **PDA‐Pt‐CD@RuFc** or **PDA** (50 µg mL^−1^) to the buffered solutions (pH = 6.5 or 7.4) containing H_2_O_2_ (30 × 10^−3^ m) at 37 °C. After incubation for different time intervals, the mixture was centrifuged and the UV/vis spectra of the residual H_2_O_2_ were recorded. The concentration was calculated from the calibration curve of absorbance at 240 nm.

*Production of O_2_ from the Reaction between H_2_O_2_ with NPs*: The production of O_2_ by the reaction of H_2_O_2_ (30 × 10^−3^ m) with the NPs (50 µg mL^−1^) or the catalase (2 U mL^−1^) at 37 °C was observed. The pictures were taken after the reaction proceeded for 30 min. O_2_ produced by the catalytic reaction was also detected by EPR measurement of the oxygen‐sensitive spin‐label probe CTPO. CTPO (0.1 × 10^−3^ m) was incubated with H_2_O_2_ at different concentrations in degassed buffered solutions (pH 6.5 or 7.4) for 15 min. After the addition of NPs (50 µg mL^−1^) or the catalase (2 U mL^−1^), the mixtures were incubated for different time intervals at room temperature. The EPR spectra were measured on a Bruker A300 X‐band EPR spectrometer (modulation amplitude, 1 G; sweep width, 100 G; microwave power, 20 mW; time constant, 163.84 ms).

*Photo‐Fenton Reaction Measurement*: The probe DMPO was used to detect the •OH produced from the Fenton reaction between **RuFc** and H_2_O_2_. DMPO (50 × 10^−3^ m) was incubated with H_2_O_2_ (30 × 10^−3^ m) and **RuFc** (50 × 10^−6^ m) in the presence or absence of light irradiation (450 nm, 20 mW cm^−2^, 3 min) at room temperature in a buffered solution (pH = 6.5 or 7.4). Then the EPR signals of DMPO/•OH were measured as described before.

*Photosensitize Generation of ^1^O_2_*: **RuFc** was incubated with ABDA (100 × 10^−6^ m) in the presence or absence of H_2_O_2_ (100 × 10^−6^ m) in buffered solutions at different pH (6.5 and 7.4). Then the mixtures were irradiated at 450 nm laser (20 W cm^−2^) for different periods of time. The characteristic UV/vis absorption spectra of ABDA were measured to determine the generation of ^1^O_2_. [Ru(bpy)_3_]Cl_2_ (bpy = 2, 2′‐bipyridine) was used as the standard (quantum yields for ^1^O_2_ production: Φ_∆_ = 0.18 in H_2_O).^45^

## Conflict of Interest

The authors declare no conflict of interest.

## Supporting information

Supplementary

## References

[1] D. E. Dolmans , D. Fukumura , R. K. Jain , Nat. Rev. Cancer 2003, 3, 380. 12724736 10.1038/nrc1071

[2] S. S. Lucky , K. C. Soo , Y. Zhang , Chem. Rev. 2015, 115, 1990. 25602130 10.1021/cr5004198

[3] a) S. Monro , K. L. Colon , H. Yin , J. Roque III , P. Konda , S. Gujar , R. P. Thummel , L. Lilge , C. G. Cameron , S. A. McFarland , Chem. Rev. 2019, 119, 797; 30295467 10.1021/acs.chemrev.8b00211 PMC6453754

[4] a) H. Huang , B. Yu , P. Zhang , J. Huang , Y. Chen , G. Gasser , L. Ji , H. Chao , Angew. Chem., Int. Ed. 2015, 54, 14049; 10.1002/anie.201507800 26447888

[5] S. Chakrabortty , B. K. Agrawalla , A. Stumper , N. M. Vegi , S. Fischer , C. Reichardt , M. Kogler , B. Dietzek , M. Feuring‐Buske , C. Buske , S. Rau , T. Weil , J. Am. Chem. Soc. 2017, 139, 2512. 28097863 10.1021/jacs.6b13399 PMC5588099

[6] a) C. S. Burke , A. Byrne , T. E. Keyes , J. Am. Chem. Soc. 2018, 140, 6945; 29767962 10.1021/jacs.8b02711

[7] K. Lou , J. F. Lovell , Chem. Commun. 2014, 50, 3231. 10.1039/c3cc49171d 24522513

[8] T. Wang , N. Zabarska , Y. Wu , M. Lamla , S. Fischer , K. Monczak , D. Y. Ng , S. Rau , T. Weil , Chem. Commun. 2015, 51, 12552. 10.1039/c5cc03473f 26153573

[9] J. P. Liu , C. Zhang , T. W. Rees , L. B. Ke , L. N. Ji , H. Chao , Coord. Chem. Rev. 2018, 363, 17.

[10] J. N. Liu , W. Bu , J. Shi , Chem. Rev. 2017, 117, 6160. 28426202 10.1021/acs.chemrev.6b00525

[11] a) C. H. Chang , J. Qiu , D. O'Sullivan , M. D. Buck , T. Noguchi , J. D. Curtis , Q. Y. Chen , M. Gindin , M. M. Gubin , G. J. W. van der Windt , E. Tonc , R. D. Schreiber , E. J. Pearce , E. L. Pearce , Cell 2015, 162, 1229; 26321679 10.1016/j.cell.2015.08.016 PMC4864363

[12] a) T. P. Szatrowski , C. F. Nathan , Cancer Res. 1991, 51, 794; 1846317

[13] M. De Palma , D. Biziato , T. V. Petrova , Nat. Rev. Cancer 2017, 17, 457. 28706266 10.1038/nrc.2017.51

[14] X. Li , N. Kwon , T. Guo , Z. Liu , J. Yoon , Angew. Chem., Int. Ed. 2018, 57, 11522. 10.1002/anie.201805138 29808948

[15] a) H. Chen , J. Tian , W. He , Z. Guo , J. Am. Chem. Soc. 2015, 137, 1539; 25574812 10.1021/ja511420n

[16] I. Moen , L. E. Stuhr , Targeted Oncol. 2012, 7, 233. 10.1007/s11523-012-0233-x PMC3510426 23054400

[17] a) Y. Liu , Y. Jiang , M. Zhang , Z. Tang , M. He , W. Bu , Acc. Chem. Res. 2018, 51, 2502; 30234960 10.1021/acs.accounts.8b00214

[18] a) Z. Shen , T. Liu , Y. Li , J. Lau , Z. Yang , W. Fan , Z. Zhou , C. Shi , C. Ke , V. I. Bregadze , S. K. Mandal , Y. Liu , Z. Li , T. Xue , G. Zhu , J. Munasinghe , G. Niu , A. Wu , X. Chen , ACS Nano 2018, 12, 11355; 30375848 10.1021/acsnano.8b06201

[19] a) E. Villemin , Y. C. Ong , C. M. Thomas , G. Gasser , Nat. Rev. Chem. 2019, 3, 261;

[20] F. Zapata , A. Caballero , A. Espinosa , A. Tarraga , P. Molina , Dalton Trans. 2009, 2009, 3900. 10.1039/b902055c 19440587

[21] R. B. Elmes , K. N. Orange , S. M. Cloonan , D. C. Williams , T. Gunnlaugsson , J. Am. Chem. Soc. 2011, 133, 15862. 21923121 10.1021/ja2061159

[22] S. Fery‐Forgues , B. Delavaux‐Nicot , J. Photochem. Photobiol., A 2000, 132, 137.

[23] a) Y. L. Liu , K. L. Ai , J. H. Liu , M. Deng , Y. Y. He , L. H. Lu , Adv. Mater. 2013, 25, 1353; 23280690 10.1002/adma.201204683

[24] X.‐S. Wang , J.‐Y. Zeng , M.‐K. Zhang , X. Zeng , X.‐Z. Zhang , Adv. Funct. Mater. 2018, 28, 1801783.

[25] a) J. X. Zhang , H. L. Sun , P. X. Ma , ACS Nano 2010, 4, 1049; 20112968 10.1021/nn901213a PMC2835570

[26] C. M. Hessel , V. P. Pattani , M. Rasch , M. G. Panthani , B. Koo , J. W. Tunnell , B. A. Korgel , Nano Lett. 2011, 11, 2560. 21553924 10.1021/nl201400z PMC3111000

[27] E. S. Lee , Z. Gao , Y. H. Bae , J. Controlled Release 2008, 132, 164. 10.1016/j.jconrel.2008.05.003 PMC2695946 18571265

[28] a) J. T. Hou , W. X. Ren , K. Li , J. Seo , A. Sharma , X. Q. Yu , J. S. Kim , Chem. Soc. Rev. 2017, 46, 2076; 28317979 10.1039/c6cs00719h

[29] L. C. Liu , L. L. Rui , Y. Gao , W. A. Zhang , Polym. Chem. 2015, 6, 1817.

[30] Y. H. Lin , J. S. Ren , X. G. Qu , Acc. Chem. Res. 2014, 47, 1097. 24437921 10.1021/ar400250z

[31] K. M. Comerford , T. J. Wallace , J. Karhausen , N. A. Louis , M. C. Montalto , S. P. Colgan , Cancer Res. 2002, 62, 3387. 12067980

[32] J. S. Armstrong , K. K. Steinauer , B. Hornung , J. M. Irish , P. Lecane , G. W. Birrell , D. M. Peehl , S. J. Knox , Cell Death Differ. 2002, 9, 252. 11859408 10.1038/sj.cdd.4400959

[33] S. Salvioli , A. Ardizzoni , C. Franceschi , A. Cossarizza , FEBS Lett. 1997, 411, 77. 9247146 10.1016/s0014-5793(97)00669-8

[34] P. O. Seglen , P. B. Gordon , Proc. Natl. Acad. Sci. U. S. A. 1982, 79, 1889. 6952238 10.1073/pnas.79.6.1889 PMC346086

[35] A. Degterev , J. L. Maki , J. Yuan , Cell Death Differ. 2013, 20, 366. 23197295 10.1038/cdd.2012.133 PMC3554332

[36] S. Sperandio , I. de Belle , D. E. Bredesen , Proc. Natl. Acad. Sci. U. S. A. 2000, 97, 14376. 11121041 10.1073/pnas.97.26.14376 PMC18926

[37] M. Garcia‐Calvo , E. P. Peterson , B. Leiting , R. Ruel , D. W. Nicholson , N. A. Thornberry , J. Biol. Chem. 1998, 273, 32608. 9829999 10.1074/jbc.273.49.32608

[38] a) H. K. Eltzschig , P. Carmeliet , N. Engl. J. Med. 2011, 364, 656; 21323543 10.1056/NEJMra0910283 PMC3930928

[39] G. L. Semenza , Cell 2012, 148, 399. 22304911 10.1016/j.cell.2012.01.021 PMC3437543

[40] D. J. Hicklin , L. M. Ellis , J. Clin. Oncol. 2005, 23, 1011. 15585754 10.1200/JCO.2005.06.081

[41] G. Finak , N. Bertos , F. Pepin , S. Sadekova , M. Souleimanova , H. Zhao , H. Chen , G. Omeroglu , S. Meterissian , A. Omeroglu , M. Hallett , M. Park , Nat. Med. 2008, 14, 518. 18438415 10.1038/nm1764

[42] M. Yamashita , T. Ogawa , X. Zhang , N. Hanamura , Y. Kashikura , M. Takamura , M. Yoneda , T. Shiraishi , Breast Cancer 2012, 19, 170. 20978953 10.1007/s12282-010-0234-5

[43] C. A. Puckett , J. K. Barton , Biochemistry 2008, 47, 11711. 18855428 10.1021/bi800856t PMC2747514

[44] X. L. Zhao , M. J. Han , A. G. Zhang , K. Z. Wang , J. Inorg. Biochem. 2012, 107, 104. 22178672 10.1016/j.jinorgbio.2011.10.007

[45] J. M. Wessels , C. S. Foote , W. E. Ford , M. A. J. Rodgers , Photochem. Photobiol. 1997, 65, 96. 9066289 10.1111/j.1751-1097.1997.tb01883.x

