# Supplementary material for: A Tailored Multifunctional Anticancer Nanodelivery System for Ruthenium‐Based Photosensitizers: Tumor Microenvironment Adaption and Remodeling
Source: Adv Sci (Weinh). 2019 Nov 25;7(1):1901992. doi: 10.1002/advs.201901992 (PMC6947499; doi:10.1002/advs.201901992)
Supplement: Supplementary file 1 — Supplementary [file ADVS-7-1901992-s001.pdf]

Copyright WILEY-VCH Verlag GmbH & Co. KGaA, 69469 Weinheim, Germany, 2019.

ADVANCED SCIENCE
Open Access

Supporting Information

for *Adv. Sci.,* DOI: 10.1002/advs.201901992

A Tailored Multifunctional Anticancer Nanodelivery System for Ruthenium-Based Photosensitizers: Tumor Microenvironment Adaption and Remodeling

Jin-Hao Liang, Yue Zheng, Xiao-Wen Wu, Cai-Ping Tan, Liang-Nian Ji, and Zong-Wan Mao**

Supporting Information

A Tailored Multifunctional Anticancer Nanodelivery System for Ruthenium-Based Photosensitizers: Tumor Microenvironment Adaption and Remodelling

Jin-Hao Liang,[†] *Yue Zheng,*[†] *Xiao-Wen Wu, Cai-Ping Tan,*[*] *Liang-Nian Ji and Zong-Wan Mao*[*]

1. Supporting Experimental Methods

Characterization of the Nanoparticles (NPs)**:** Fourier transform infrared (FT-IR) spectrometry was performed on a Bruker Vector-22 infrared spectrometer (Germany). The morphology of **PDA**, **PDA-Pt**, **PDA-Pt-CD** and **PDA-Pt-CD@RuFc** was observed by transmission electron microscopy (TEM, JEOL, JEM1400Plus 120 kv, Japan and FEI Tecnai[TM], F30 300 kv, USA). The NPs were dropped onto a copper grids coated carbon membrane, and dried in the air. Dynamic light scattering (DLS) and zeta potential were tested by a Zetasizer Nano instrument (EliteSizer, Bruker). TEM in conjunction with energy-dispersive X-ray spectroscopy (TEM-EDS) elemental maps were analyzed by an Energy Dispersive X-ray Spectrometer (FEI Tecnai G2 Spirit, Holland). UV/Vis spectra were recorded on a Varian Cary 300 spectrophotometer (USA). The fluorescence emission spectra were obtained using an Edinburgh FLS 920 Spectrometer (UK).

Drug Loading and Releasing Measurement: The load capacity of **RuFc** on **PDA-Pt-CD@RuFc** is calculated by subtracting the absorption of **PDA-Pt@CD-PEG** at 465 nm. The release of **RuFc** from **PDA-Pt-CD@RuFc** under

different stimuli was determined by measuring the content of **RuFc** in the supernatant calculated from the absorption at 465 nm after centrifugation. In order to study the effect of pH values, **PDA-Pt-CD@RuFc** was incubated in PBS (pH = 6.5 or 7.4) at room temperature for 1 h. For the photothermal release, **PDA-Pt-CD@RuFc** in PBS (pH = 6.5 or 7.4) was irradiated with an 808 laser (1 W cm^{-2}). For H_2O_2-triggered release, **PDA-Pt-CD@RuFc** in PBS (pH = 6.5 or 7.4) was treated with H_2O_2 (100 mM) at 1 h.

Inductively Coupled Plasma-Mass Spectrometry (ICP-MS: A549 cells were incubated with containing **RuFc** (10 μM) or **PDA-Pt-CD-PEG** (25 μg mL^{-1}) or **PDA-Pt-CD@RuFc** (25 μg mL^{-1}). After 12 h incubation, the cells were collected in PBS (1 mL). Mitochondria were isolated from cells using the mitochondria isolation kit (89874, Thermo Scientific). Nuclear and cytosolic fractions were separated using a nucleoprotein extraction kit (C500009, Sangon Biotech, China). The radio immunoprecipitation assay (RIPA) buffer was used for cell lyses. The protein concentration of each fraction was determined by the bicinchoninic acid (BCA) assay. The mixtures containing HNO_3 (65%, 500 μL) and lysates were incubated at room temperature for 24 h to digest entirely. Then, Milli-Q water (containing 10 ppb indium as internal standard) was added into the solution to a final volume of 10 mL. The concentration of ruthenium or platinum was measured using the XSERIES 2 ICP-MS.

Photothermal Activity of ***PDA-Pt-CD@RuFc***: The aqueous solution of **PDA-Pt** and **PDA-Pt-CD@RuFc** (50, 100 and 200 μg mL^{-1}) was placed in a quartz cuvette and

irradiated with 808 nm laser (1 W cm^{-2}) for 10 min. The temperature was recorded by a thermal temperature probe. Millipore water was used as the control group.

Cell Lines and Culture Conditions: 4T1, MB-MDA-231, HeLa, LO2 and RAW 264.7 cells were obtained from Experimental Animal Center of Sun Yat-Sen University (Guangzhou, China). Cells were maintained in 1640 medium containing 10% FBS, streptomycin (100 μg mL^{-1}) and penicillin (100 U mL^{-1}). The cells were cultured in a humidified incubator in an atmosphere of 5% CO_2 and 95% air at 37 °C.

One-Photon PDT (OPPDT) and PTT Synergistic Treatment: The evaluation of the combined PDT-PTT Therapeutic effects of NPs was carried out ad described before.[1] Cells were cultured in hypoxia (1%) or normoxia (21%) atmosphere. First, a series of preliminary experiments were used to select the best treatment conditions for combination therapy. The standard is that light does not damage cells, and the effect of one treatment is not significantly masked by another. For the PTT groups, the experimental conditions we chose were 17 mW cm^{-2} and 1 min irradiation with an 808 nm NIR laser. For the PDT groups, the experimental conditions were 20 mW cm^{-2} and 2 min irradiation with a 450 nm laser. For the combined PTT-PDT treatment, cells were serially treated with the 808 nm laser and the 450 nm laser. Cells were treated with different concentrations of **PDA-Pt-CD@RuFc** for 24 h. After light irradiation, the cells were further incubated for another 24 h and the cell viability was determined by CellTiter-Glo kit (Promega).

Two-Photon PDT (TPPDT) and PTT Synergistic Treatment: 4T1 cells were treated with **PDA-Pt-CD@RuFc** (50 μg mL^{-1}) for 24 h and then irradiated with one or two

lasers (808 nm: 1 W cm^{-2}, 10 min; 810 nm (two-photon laser): 100 mW, 20 s, 80 MHz, 100 fs). After incubated for another 12 h, the cells were staining with calcein AM (2 μM) solutions for 30 min (Invitrogen, USA). Then images were taken on a confocal microscope (10 × objective) (LSM 810, Carl Zeiss, Göttingen, Germany). Calcein AM: λ_{ex} = 488 nm; λ_{em} = 520 ± 20 nm.

A number of 4000 diluted 4T1 cells were transferred to 1.5% agarose-coated transparent 96-well plates with 150 μL of culture media. The cells were then resuspended in culture media and formed multicellular tumor spheroids (MCTSs) aggregates approximately 300 μm in diameter after 3 days. The cell solution in the inlet was replaced with fresh cell culture media every two days to maintain the growing of MCTSs.

4T1 MCTSs of 400~500 μm diameter were treated with **PDA-Pt-CD@RuFc** (50 μM) for 24 h. Then the MCTSs were irradiated with one or two lasers (808 nm: 1 W cm^{-2}, 10 min; 810 nm (two-photon laser): 100 mW, 20 min, 80 MHz, 100 fs). After incubated for another 12 h, the cells were staining with calcein AM (2 μM) solutions for 30 min (Invitrogen, USA). Then images were taken on a confocal microscope (10 × objective) (LSM 810, Carl Zeiss, Göttingen, Germany). Calcein AM: λ_{ex} = 488 nm; λ_{em} = 520 ± 20 nm.

In order to quantitatively detect the cytotoxicity of TPPDT-PTT mediated by **PDA-Pt-CD@RuFc**, the MCTSs were irradiated with one or two lasers (808 nm: 1 W cm^{-2}, 10 min; 810 nm (two-photon laser): 100 mW, 20 min, 80 MHz, 100 fs) and incubated for another 24 h, the adenosine triphosphate (ATP) concentration was

measured using the Cell TiterGlo 3D Cell Viability kit (Promega) as we previously described.[2]

RT-qPCR Measurement of the Expression of HIF-1α and MDR1 Genes: RT-qPCR was performed as we previously described[3] using a Roche LightCycler 480 Detection System (Roche, USA) and SYBR Green I Master (Roche, USA). 4T1 cells were incubated in hypoxia (1%) or normoxia (21%) atmosphere. Briefly, 4T1 cells were treated with **PDA-Pt-CD@RuFc** (25 or 50 μg mL^{-1}) for 6 h. Total RNA is isolated using the Trizol reagent (Life technologies, USA). The synthesis of cDNA was done using the iScript cDNA synthesis kit (BIO-RAD, USA). GAPDH was used as the reference gene. The primer sequences are listed as below.

Table S1. Primer sequences for RT-qPCR.

HIF-1α-F	CCACAGGACAGTACAGGATG
HIF-1α-R	TCAAGTCGTGCTGAATAATACC
MDR1-F	GAGGAAGACATGACCAGGTATGC
MDR1-R	GTGTTAAGCTCCCCAACATCG

Cellular ROS Detection: Intracellular ROS production were measured in the absence or presence of light using DCFH-DA as the sensor. The detailed staining procedures were described before.[3] Briefly, 4T1 cells were incubated with **PDA-Pt-CD@RuFc** NPs (25 or 50 μg mL^{-1}) for 24 h. The cells were then stained and observed by confocal microscopy. The experiment was done under both the hypoxic (1% O_2) and normoxic (21% O_2) conditions. Irradiation conditions: 450 nm, 17 mW cm^{-2}, 1 min.

Measurement of Cathepsin B Activity: Cathepsin B activity was detected using the Magic Red MR-$(RR)_2$ assay following the manufacturer's instructions according to our previous reports.[4] Briefly, 4T1 cells were treated with **PDA-Pt-CD@RuFc** (50 μg mL^{-1}) for 24 h. The cells were then irradiated with a 450 nm laser (17 mW cm^{-2}, 1 min) and an 808 nm laser (1 W cm^{-2}, 10 min). The cells were then stained with Magic Red MR-$(RR)_2$ at 37 °C for 1 h and observed by confocal microscopy. λ_{ex} = 543 nm; λ_{em} = 630 ± 20 nm.

Analysis of MMP: MMP was detected by JC-1 staining as we previously reported.[3] Briefly, 4T1 cells were treated with **PDA-Pt-CD@RuFc** (50 μg mL^{-1}) for 24 h. The cells were then irradiated with a 450 nm laser (17 mW cm^{-2}, 1 min) and an 808 nm laser (1 W cm^{-2}, 10 min). Then the cells were stained with JC-1 and analyzed by confocal microscopy.

Annexin V Staining Assay: The assay was performed according to the manufacturer's (Sigma Aldrich, USA) protocol as we previously reported.[3] 4T1 cells were treated with **PDA-Pt-CD@RuFc** NPs (50 μg mL^{-1}) for 24 h. The cells were then irradiated with a 450 nm laser (17 mW cm^{-2}, 1 min) and an 808 nm laser (1 W cm^{-2}, 10 min). The cells were stained and analyzed immediately by confocal microscopy and flow cytometry.

Caspase 3/7 Activity Assay: The activation of caspase 3/7 was determined using the Caspase-Glo 3/7 assay kit (Promega).[3] 4T1 cells were treated with **PDA-Pt-CD@RuFc** NPs (25 or 50 μg mL^{-1}) for 24 h. The cells were then irradiated with a 450 nm laser (17 mW cm^{-2}, 1 min) and an 808 nm laser (1 W cm^{-2}, 10 min).

After the addition of the assay reagents, the relative luminescent units (RLU) were detected with a microplate reader (Infinite F200, Tecan, Switzerland).

Intracellular ATP Levels: Intracellular ATP levels was determined using the CellTiter-Glo kit (Promega).[3] Briefly, 4T1 cells were treated with **PDA-Pt-CD@RuFc** NPs (25 or 50 μg mL^{-1}) for 24 h. The cells were then irradiated with a 450 nm laser (17 mW cm^{-2}, 1 min) and an 808 nm laser (1 W cm^{-2}, 10 min). After the addition of the assay reagents, the relative luminescent units (RLU) were detected with a microplate reader (Infinite F200, Tecan, Switzerland). The results are averaged among 3 replicates, and have been normalized by the values obtained on untreated control cells. Error bars represent the standard deviation.

Impact of Inhibitors on Cell Death: Different inhibitors were used to investigate the cell death modes induced by the **PDA-Pt-CD@RuFc** NPs. The cells were treated with **PDA-Pt-CD@RuFc** NPs (25 or 50 μg mL^{-1}) for 24 h. The cells were then irradiated with a 450 nm laser (17 mW cm^{-2}, 1 min) and an 808 nm laser (1 W cm^{-2}, 10 min). These inhibitors were added 1 h before the irradiation. After a further incubation for 24 hours, the cell viability was measured by MTT assay. Concentrations of the inhibitors: z-VAD-FMK, 50 μM; 3-Methyladenine, 50 μM; Necrostatin-1, 100 μM; Cycloheximide, 1 μM.

Animals: All animal procedures were performed in accordance with the Guidelines for Care and Use of Laboratory Animals of Sun Yat-Sen University and the experimental protocols were approved by the Sun Yat-Sen University Animal Care and Use Committee. Female BALB/c mice with body weights of 19~21 g were obtained from

Beijing Vitalriver Experimental Animal Technology Co. Ltd. The mice were housed in individual ventilated cages at 20 ± 2 ^{o}C room temperature and 60 ± 10% relative humidity with a light/dark cycle of 12 h.

Biological Distribution and Metabolism of NPs: BALB/c mice carrying 4T1 tumors were injected with **PDA-Pt-CD@RuFc** NPs (200 μL, 1 mg mL^{-1}) intravenously. The mice were dissected 1, 2, 4 and 7 days after treatment. The main organs of the mice (n = 3) were collected and dissolved in aqua regia. The contents of ruthenium and platinum were measured by ICP-MS using the XSERIES 2 ICP-MS (Thermo Scientific, USA) as previously described.[4]

Photothermal Imaging: BALB/c mice implanted with 4T1 tumors were injected with **PDA-Pt-CD@RuFc** (200 μL, 1 mg mL^{-1}) intratumorally or intravenously. After 4 h, the mice were irradiated with an 808 nm laser (1 W cm^{-2}) for 0 min, 1 min, 3 min and 5 min. The thermal images were recorded using an infrared Fluke Ti55 Thermal Imager (Fluke Corporation, Everett, WA) camera.

Photoacoustic (PA) Imaging: BALB/c mice carrying 4T1 tumors were injected with **PDA-Pt-CD@RuFc** (200 μL, 1 mg mL^{-1}) intravenously and the images were taken at different time points. PA imaging was conducted using a Vero LAIR animal photoacoustic and ultrasound imaging system (Nexus128, USA) stimulated by an 810 nm laser.

Computed Tomograhy (CT) Imaging: BALB/c mice carrying 4T1 tumors were injected with **PDA-Pt-CD@RuFc** (100 μL, 5 mg mL^{-1}) intratumorally or intravenously. CT images were taken 30 min after intratumoral injection and 2 hours

after intravenous injection. Before imaging, the mice were injected with anesthetics intraperitoneally (4% chloral hydrate, 200 μL) for anesthetization. The CT images were captured by an Inveon Multimodality PET/CT-system for small animals (Preclinical Solutions, Siemens Healthcare Molecular Imaging, USA).

In Vivo Antitumor Activity Evaluation: BALB/c mice carrying 4T1 tumors were randomly divided into 9 groups: (i) saline control group; (ii) dark + intratumoral (i.t.) injection group; (iii) PDT + i.t. injection group; (iv) PTT + i.t. injection group; (v) PDT+ PTT + i.t. injection group; (vi) dark + intravenous (i.v.) injection group; (vii) PDT + i.v. injection group; (viii) PTT + i.v. injection group; (ix) PDT+ PTT + i.v. injection group. When tumor volume reaches 100–150 mm^3, the mice were treated with **PDA-Pt-CD@RuFc** NPs (200 μL, 1 mg mL^{-1}). The groups with i.t. injection were irradiated immediately after administration. The groups with i.v. injection were irradiated 4 h after administration. For the combined treatment groups, the treatments were carried out in the order of PDT followed by PTT. Irradiation conditions: 450 nm, 12 W cm^{-2}, 5 min; 808 nm, 1 W cm^{-2}, 3 min. The weight and tumor size of mice were recorded every two days.

H&E Staining: All groups of mice with in vivo antitumor activity evaluation were dissected. The tumor tissues and the main organs (heart, liver, spleen, lung and kidney) were fixed with paraffin and examined using H&E staining as previously described.[4] The pictures were taken by a digital microscope (Leica QWin).

Enzyme-Linked Immunosorbent Assay (ELISA) for Cytokines: For cell-based measurement, 4T1 cells and RAW 264.7 macrophages were all precultured on a

96-well plate for 24 h in 5% CO_2 at 37 °C, respectively. 4T1 cells were incubated with **PDA-Pt-CD@RuFc** NPs (50 μg mL^{-1}) for another 24 h, after that, the cells were carefully washed again with PBS for three times and 200 μL of H_2O_2 (3 mM in fresh 1640 medium) was added and incubated for 2 h. The cells only treated with nanoparticles or H_2O_2 were also conducted according to above method. The 4T1 cells without treatment served as control. After that, the supernatants of 4T1 cells were transferred to the 96-well plate to culture RAW 264.7 macrophages overnight. In addition, RAW 264.7 cultures were stimulated with LPS (100 ng mL^{-1} in RPMI 1640 medium) for 20 h. The supernatants of RAW 264.7 macrophages were harvested and used to measure proinflammatory cytokines (TNF-α) and interleukin 6 (IL-6) by kits according to manufacturer's instructions.

For the in vivo assay, 12 tumor-bearing mice were divided into 4 groups to detect sera cytokines. Each mouse was intravenously injected with: (1) PBS; (2) PBS; (3) **PDA-Pt-CD@RuFc** NPs (1 mg mL^{-1}) and (4) **PDA-Pt-CD@RuFc** NPs (1 mg mL^{-1}). At 12 h post-injection, mice in group (2) and (4) were intravenously injected into 300 μL of H_2O_2 (10 mM) or saline buffer. After 24 h, mice in above treatment schemes were collected and their blood was drawn from the eye socket, directly placed in the empty centrifuge tube, without any anticoagulant treatment, the blood gradually coagulation, a few hours after the blood clot slowly shrink and release yellow liquid called serum samples for analysis. TNF-α and IL-6 were measured by the kits according to manufacturer's instructions.

Immunohistochemical Assay: BALB/c mice bearing 4T1 tumors were randomly divided into 5 groups: (i) saline group; (ii) dark group; (iii) PDT group; (iv) PTT group; (v) PDT+ PTT group. When tumor volume reaches 100–150 mm^3, the mice were treated with **PDA-Pt-CD@RuFc** NPs (200 μL, 1 mg mL^{-1}). The mice were irradiated 4 h after administration. For the combined treatment groups, the treatments were carried out in the order of PDT followed by PTT. Irradiation conditions: 450 nm, 12 W cm^{-2}, 5 min; 808 nm, 1 W cm^{-2}, 3 min. After 24 h, the mice were dissected and the tumor tissues were fixed, embedded, sliced and stained histochemically for microscopic observation.

Immunohistochemical staining: The slices were heated to 57 °C for 0.5 h and deparaffinized with xylene, anhydrous ethanol, 95% ethanol, 80% ethanol, 70 % ethanol and distilled water serially. The retrieval of antigen was performed in EDTA antigen repair buffer (pH 9.0) at 90 °C for 1 h. Then, the slices are incubated in 3% H_2O_2 for 20 min at room temperature, and washed with PBS (pH 7.4) for 3 times. After the sections were slightly dried, they were incubated with the primary antibodies overnight at 4 °C. After washed with PBS (pH 7.4) for 3 times, the slides were incubated with the HRP-conjugated secondary antibody for 50 min. After staining with hematoxylin, the images were observed and collected under a Nikon inverted fluorescence microscope.

Immunofluorescence Staining: The specimens were preparation as described above. After incubation with the primary antibody overnight at 4 °C, the specimens were incubated with the fluorochrome-conjugated secondary antibody for 1 h at room

temperature. The nuclei were stained with DAPI, and the images were observed and collected under a Nikon inverted fluorescence microscope.

Statistical analysis: All biological experiments were performed at least twice with triplicates in each experiment. Representative results were depicted in this report and data were presented as means ± standard deviations (SD).

2. Supporting Scheme, Figures and Tables

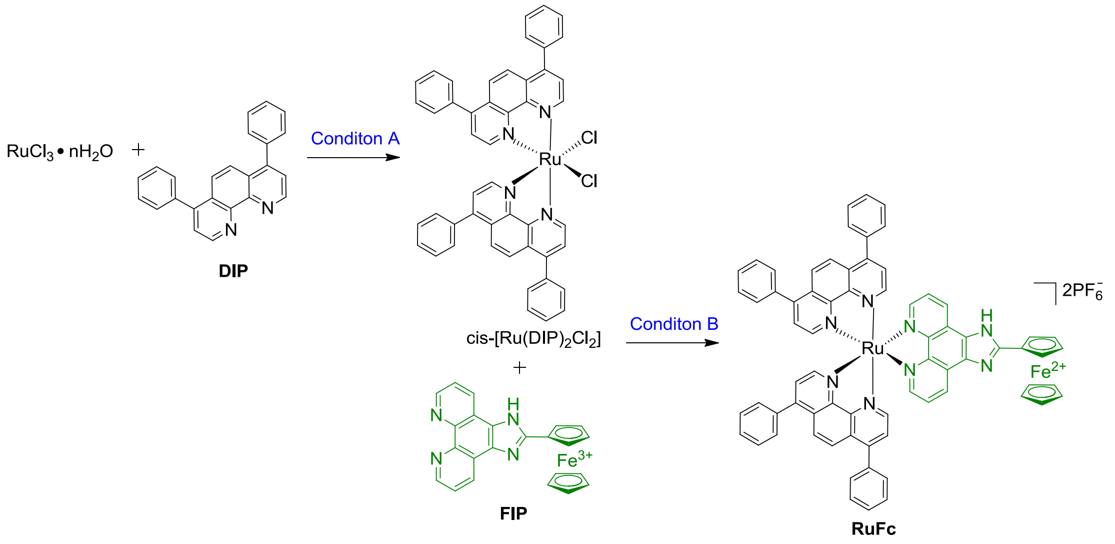

Scheme S1. The synthetic route of **RuFc**. Condition A: LiCl, DMF, reflux 8 h, N_2 Condition B: CH_3OH/H_2O (3:1), reflux 8 h, N_2.

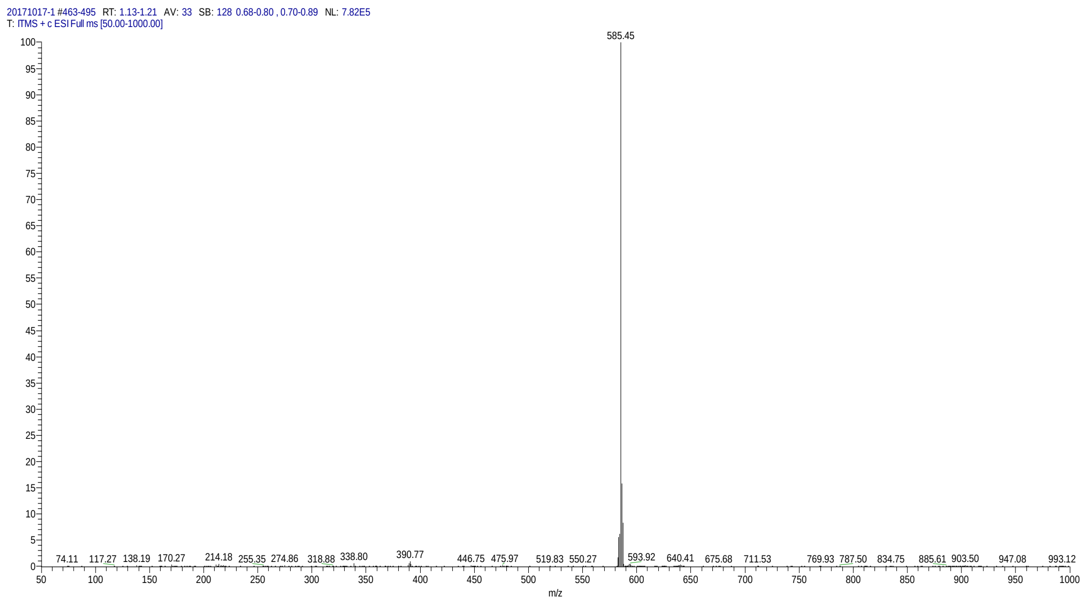

Figure S1. ESI-MS spectrum of **RuFc**.

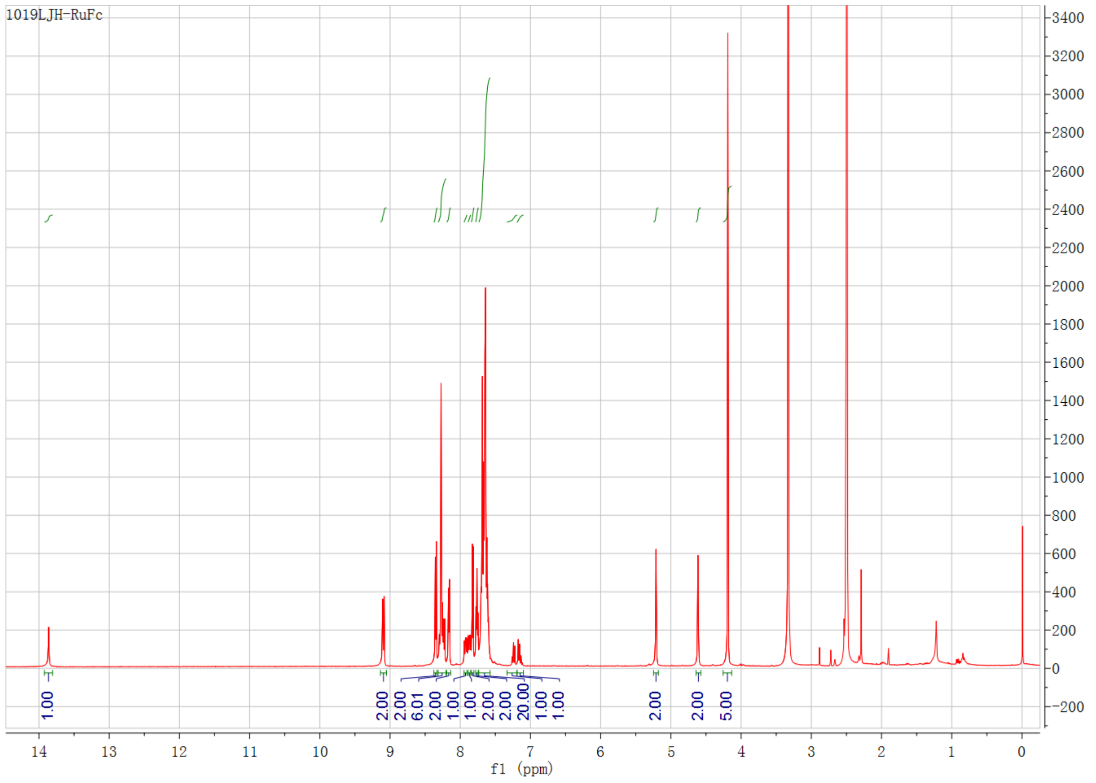

Figure S2. ^{1}HNMR spectrum of **RuFc** in DMSO-d_6.

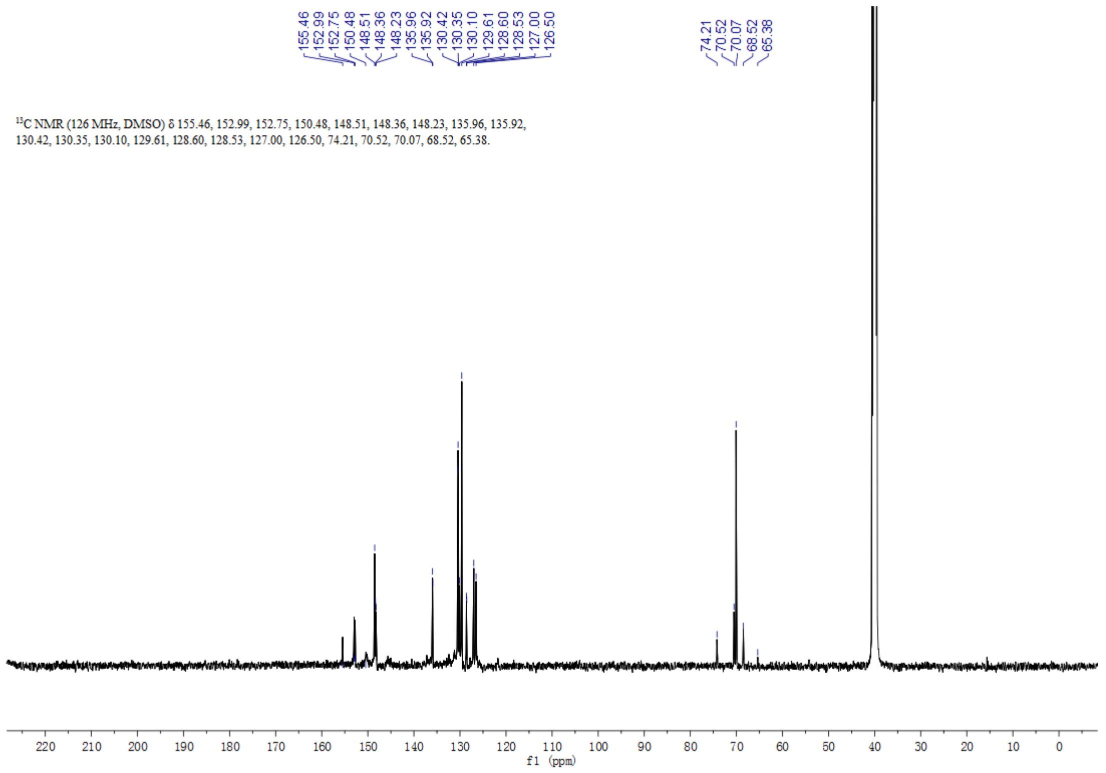

Figure S3. ^{13}C NMR spectrum of **RuFc** measured in DMSO-d_6.

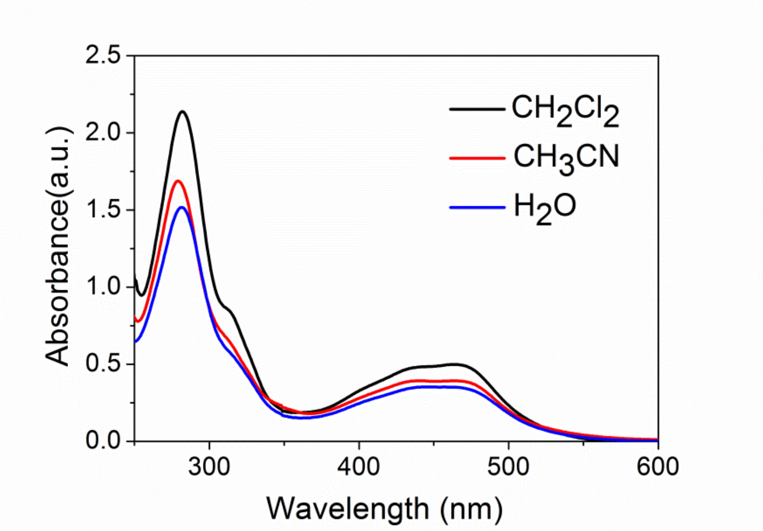

Figure S4. UV/Vis absorption spectra of **RuFc** (1×10^{-5} mol L^{-1}) in degassed CH_3CN, CH_3Cl_2 and H_2O at 298 K.

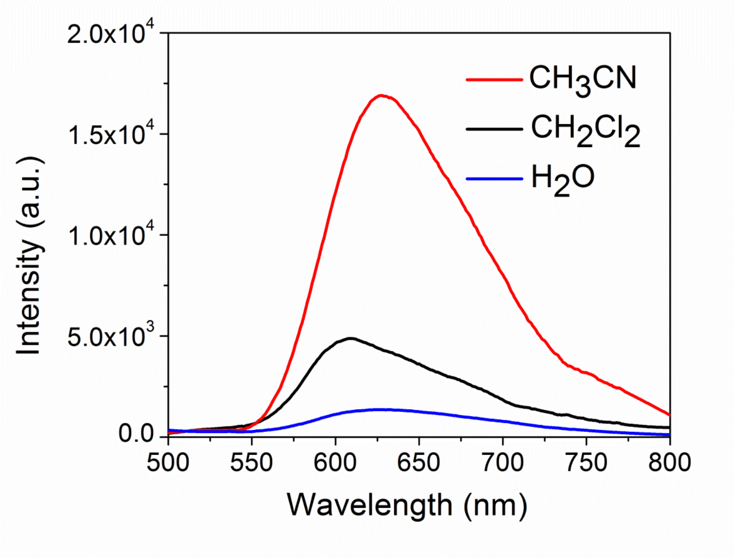

Figure S5. Emission spectra of **RuFc** (1×10^{-5} mol L^{-1}) in degassed CH_3CN, CH_3Cl_2 and H_2O at 298 K.

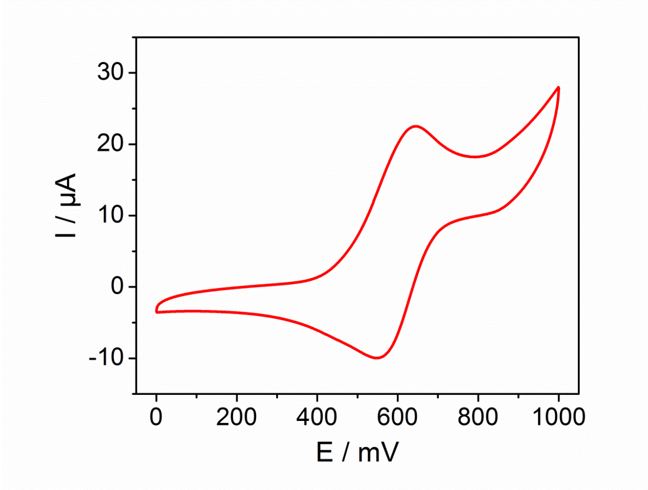

Figure S6. Cyclic voltammogram of **RuFc** (10 μM) in CH_2Cl_2.

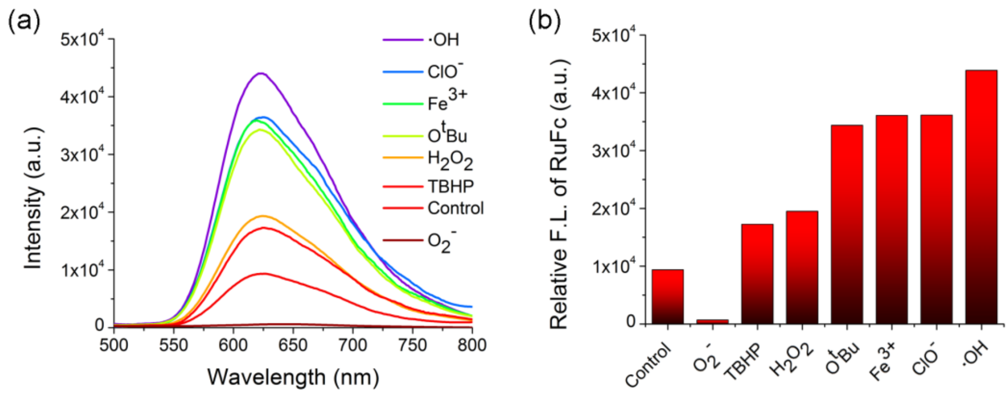

Figure S7. (a) Fluorescence responses of **RuFc** (1×10^{-5} mol L^{-1}) with ROS. (b) Relative fluorescence responses of **RuFc** (1×10^{-5} mol L^{-1}) with ROS. **RuFc** was incubated with variety of oxidants for 1 h. Oxidant concentrations: O_2^{-}: 1 mM; Tert-Butyl Hydroperoxide (TBHP), 100 μM; H_2O_2, 100 μM; O^tBu, 100 μM; Fe^{3+}, 100 μM; ClO^{-}, 100 μM; •OH, 100 μM.

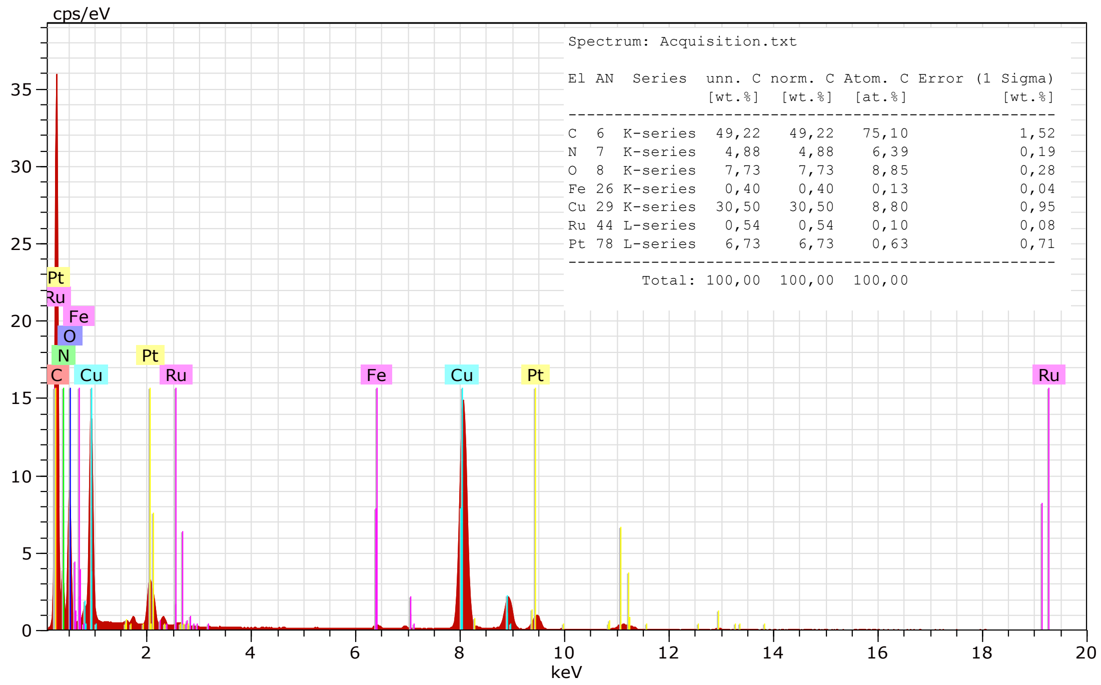

Figure S8. The EDX spectrum of **PDA-Pt-CD@RuFc** NPs.

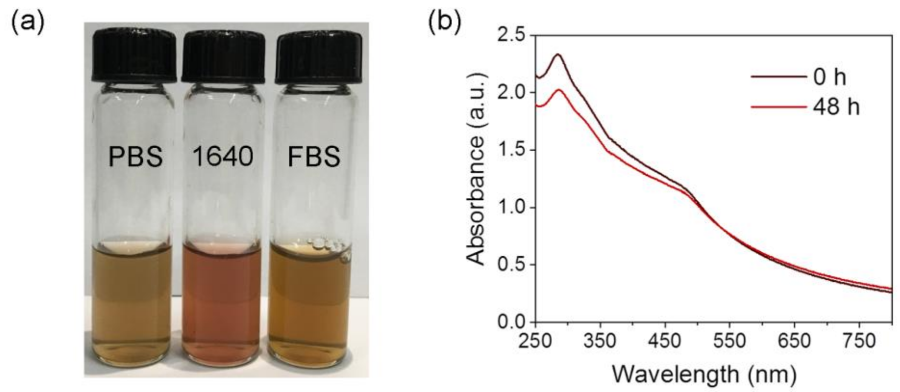

Figure S9. (a) The stability of **PDA-Pt-CD@RuFc** NPs in PBS, 1640 medium, and FBS after 2 days incubation. (b) The stability of **PDA-Pt-CD@RuFc** (50 µg mL^{-1}) in PBS measured by UV/Vis absorption measurement.

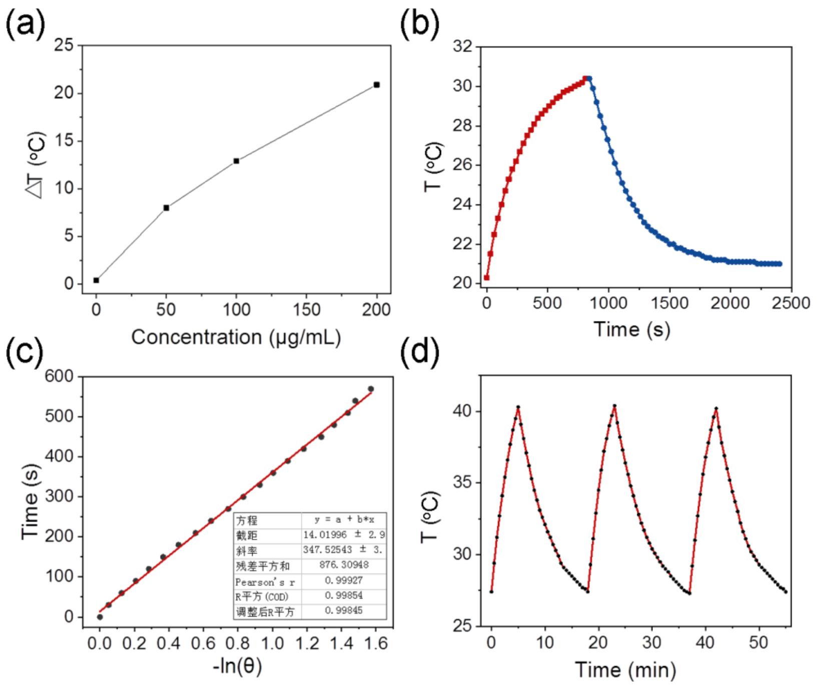

Figure S10. (a) Temperature changes of different concentrations of **PDA-Pt-CD@RuFc** NPs. The solutions were irradiated with an 808 nm laser (1 W cm^{-2}) for 10 min. Water is used as controls. (b) Photothermal effect of the irradiation of the aqueous dispersion of **PDA-Pt-CD@RuFc** NPs (100 μg mL^{-1}) with the 808 nm NIR laser (1 W cm^{-2}). The irradiation lasted for 800 s, and then the laser was turned off. (c) Plot of cooling time versus negative natural logarithm of the temperature driving force which is obtained from the cooling stage. (d) Temperature variations of **PDA-Pt-CD@RuFc** NPs (100 μg mL^{-1}) under irradiation by 808 nm laser at the power density of 1 W cm^{-2} for three cycles (5 min irradiation for each cycle).

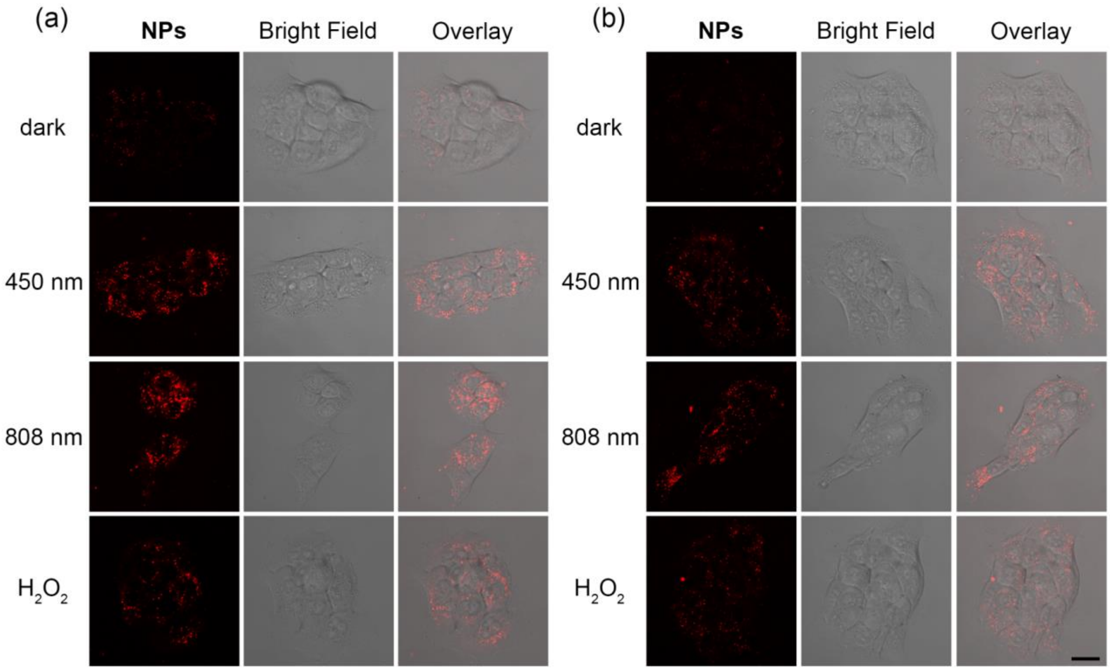

Figure S11. Drug release of **PDA-Pt-CD@RuFc** upon 450 nm laser, 808 nm laser or H_2O_2 pre-treatment in the (a) pH 5.0 and (b) pH 7.4. The cells were pre-treated with H_2O_2 (3 mM) for 45 min. Irradiation conditions: 450 nm, 17 mW cm^{-2}, 1 min; 808 nm, 1 W cm^{-2}, 10 min. Scale bar: 20 μm.

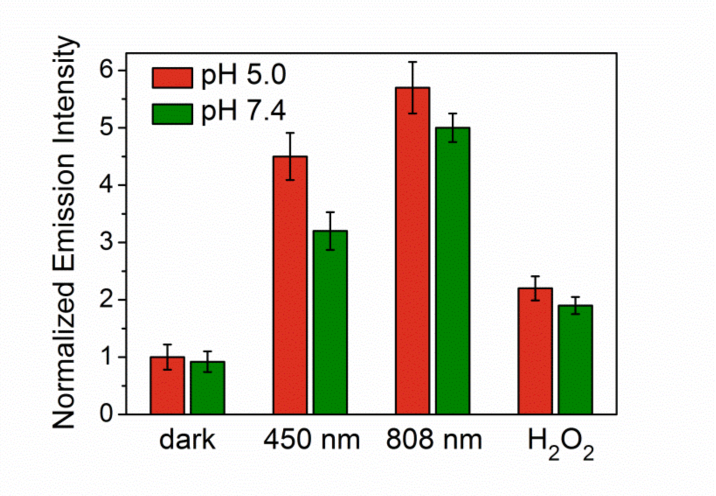

Figure S12. Comparison of the intracellular phosphorescence intensity of **PDA-Pt-CD@RuFc** upon 450 nm laser, 808 nm laser or H_2O_2 pre-treatment in the pH 5.0 and pH 7.4. Data are expressed as the mean ± SD (standard deviations). Number of cells: 10.

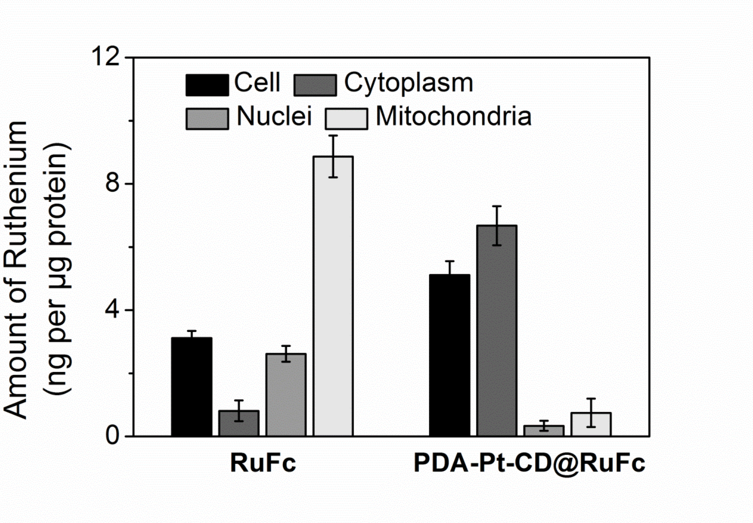

Figure 13. Distribution of **RuFc** and **PDA-Pt-CD@RuFc** in cellular compartments of 4T1 cells measured by ICP-MS using ^{101}Ru as the standard. The cells were incubated with **RuFc** (10 μM) or **PDA-Pt-CD@RuFc** (25 μg mL^{-1}) for 12 h.

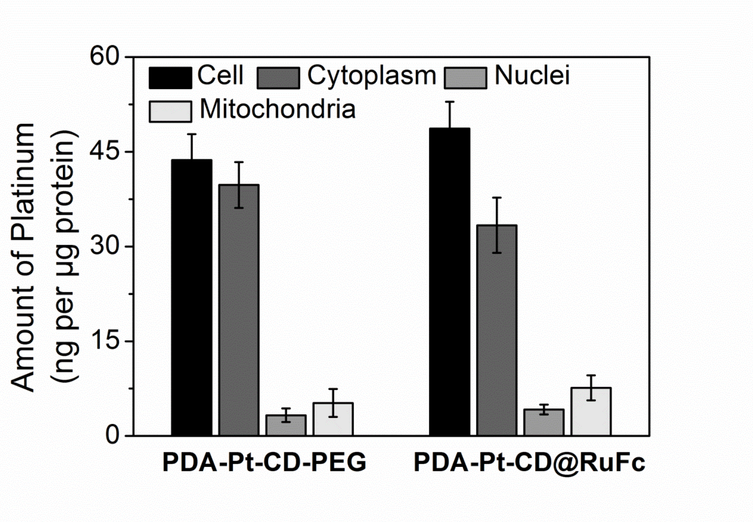

Figure S14. Distribution of **PDA-Pt-CD-PEG** and **PDA-Pt-CD@RuFc** in cellular compartments of 4T1 cells measured by ICP-MS using ^{195}Pt as the standard. The cells were incubated with **PDA-Pt-CD-PE** (25 μg mL^{-1}) or **PDA-Pt-CD@RuFc** (25 μg mL^{-1}) for 12 h.

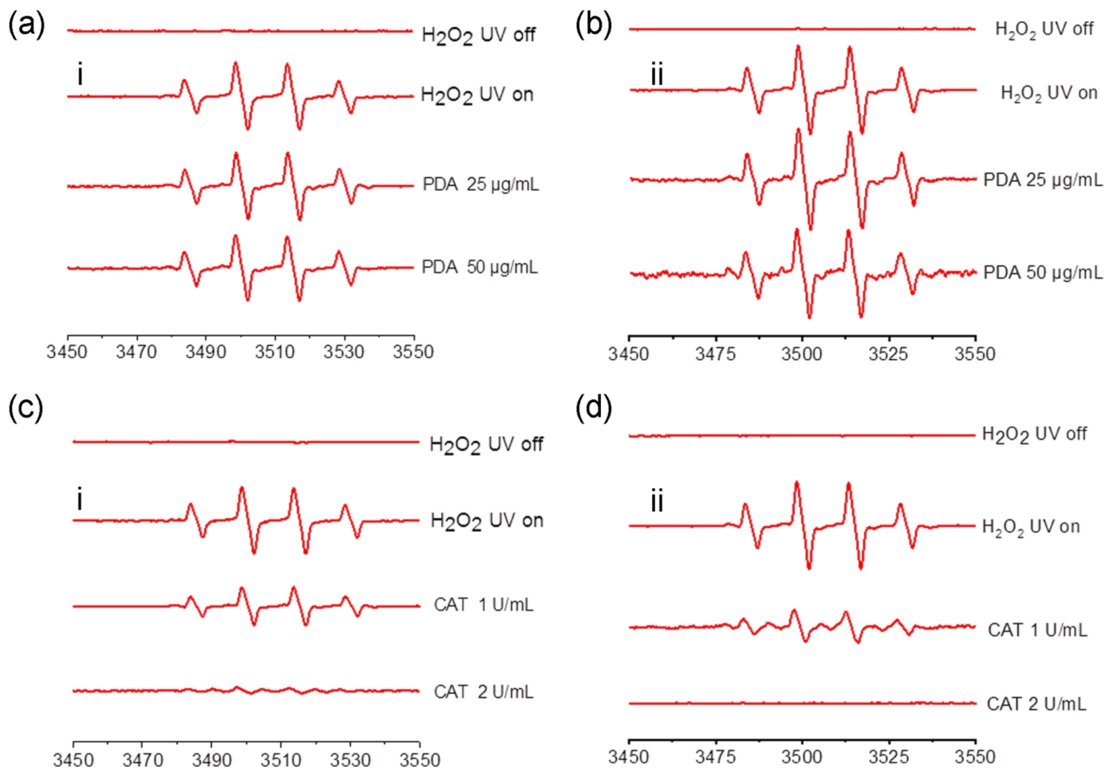

Figure S15. Effect of **PDA** (a and b) and catalase (CAT, c and d) on •OH production at different concentrations in a H_2O_2/UV system at pH 6.5 (i) and pH 7.4 (ii).

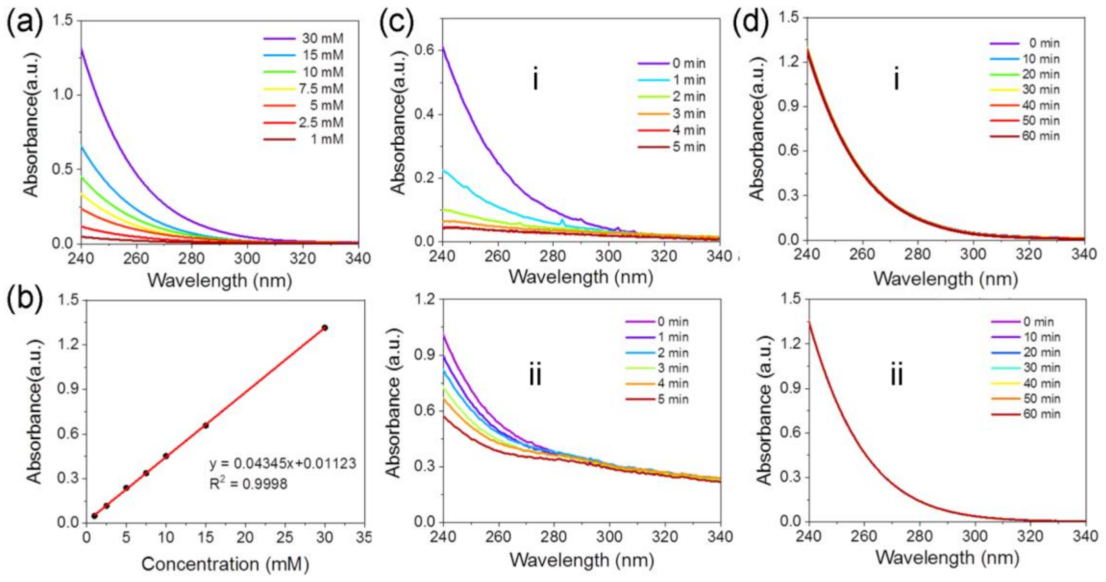

Figure S16. Decomposition of H_2O_2 by NPs. (a) The UV-Vis spectra of H_2O_2 measured at different concentrations; (b) The calibration curve of absorbance at 240 nm and the concentrations of H_2O_2. The UV/Vis spectra of remainder H_2O_2 were recorded after the reaction of H_2O_2 (30 mM) with (c) **PDA-Pt-CD@RuFc** (100 ug mL^{-1}) and (d) **PDA** (100 ug mL^{-1}) for different times at pH 6.5 (i) and pH 7.4 (ii).

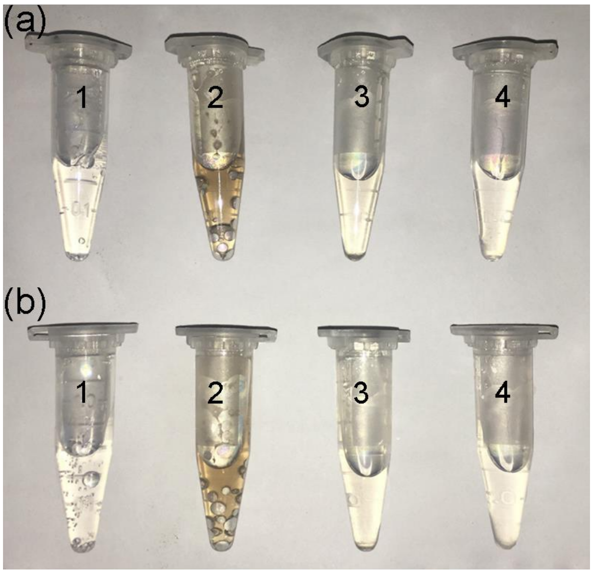

Figure S17. A photo of tubes containing different solutions at 37 °C (a: pH 6.5; b: pH 7.4). (1) catalase + H_2O_2 (30 mM); (2) **PDA-Pt-CD@RuFc** (50 ug mL^{-1}) + H_2O_2 (30 mM); (3) **PDA** (50 ug mL^{-1}) + H_2O_2 (30 mM); (4) H_2O_2 (30 mM).

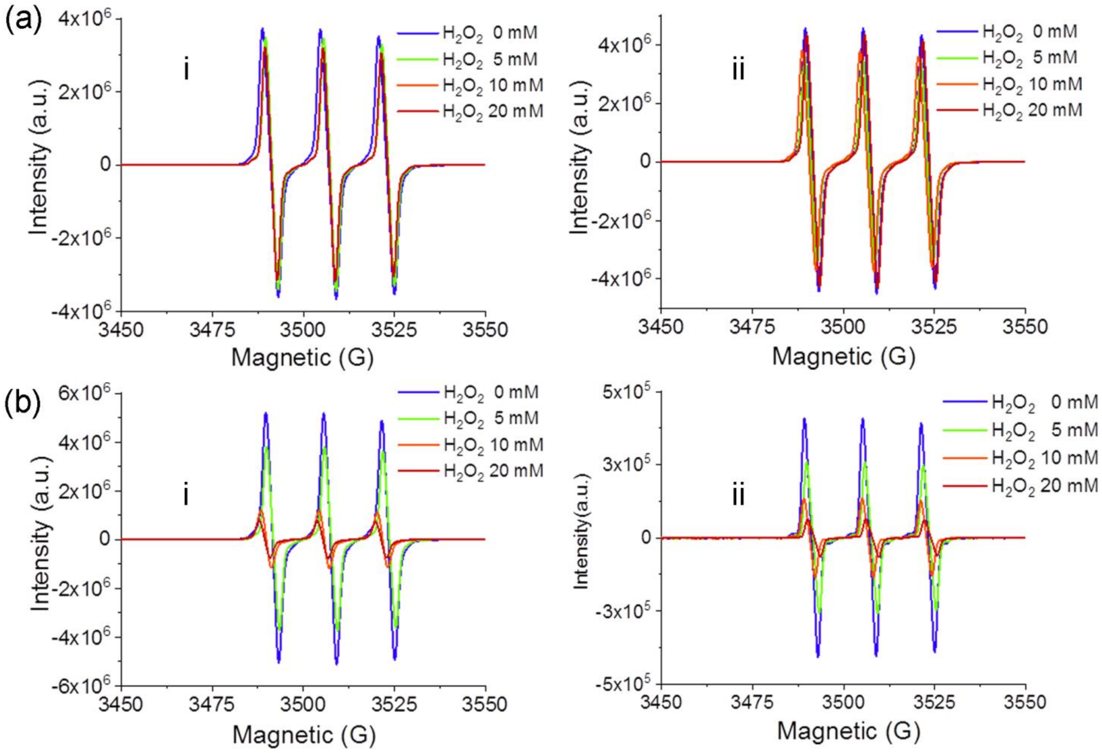

Figure S18. Effect of (a) **PDA** (50 μg mL^{-1}) and (b) catalase (2 U mL^{-1}) on O_2 production detected by the probe CTPO (0.1 mM) in the H_2O_2/UV system at pH 6.5 (i) and pH 7.4 (ii).

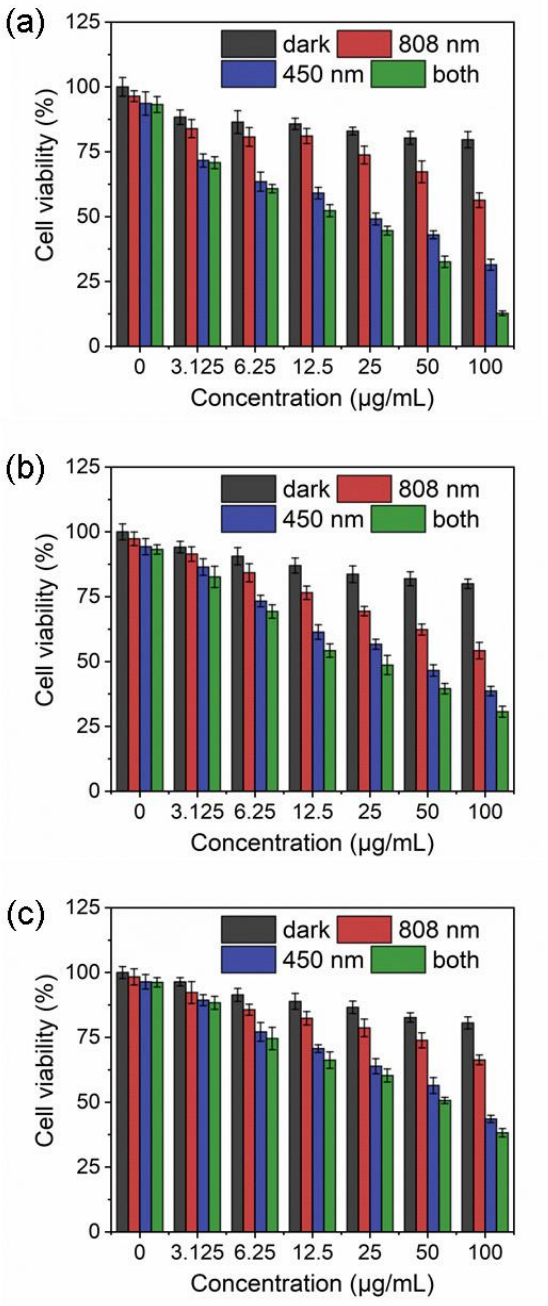

Figure S19. *In vitro* combined PDT-PTT activities of **PDA-Pt-CD@RuFc** NPs measured on MB-MDA-231 (a), HeLa (b) and LO2 (c) cells. Irradiation conditions: 450 nm, 17 mW cm^{-2}, 1 min; 808 nm, 1 W cm^{-2}, 10 min.

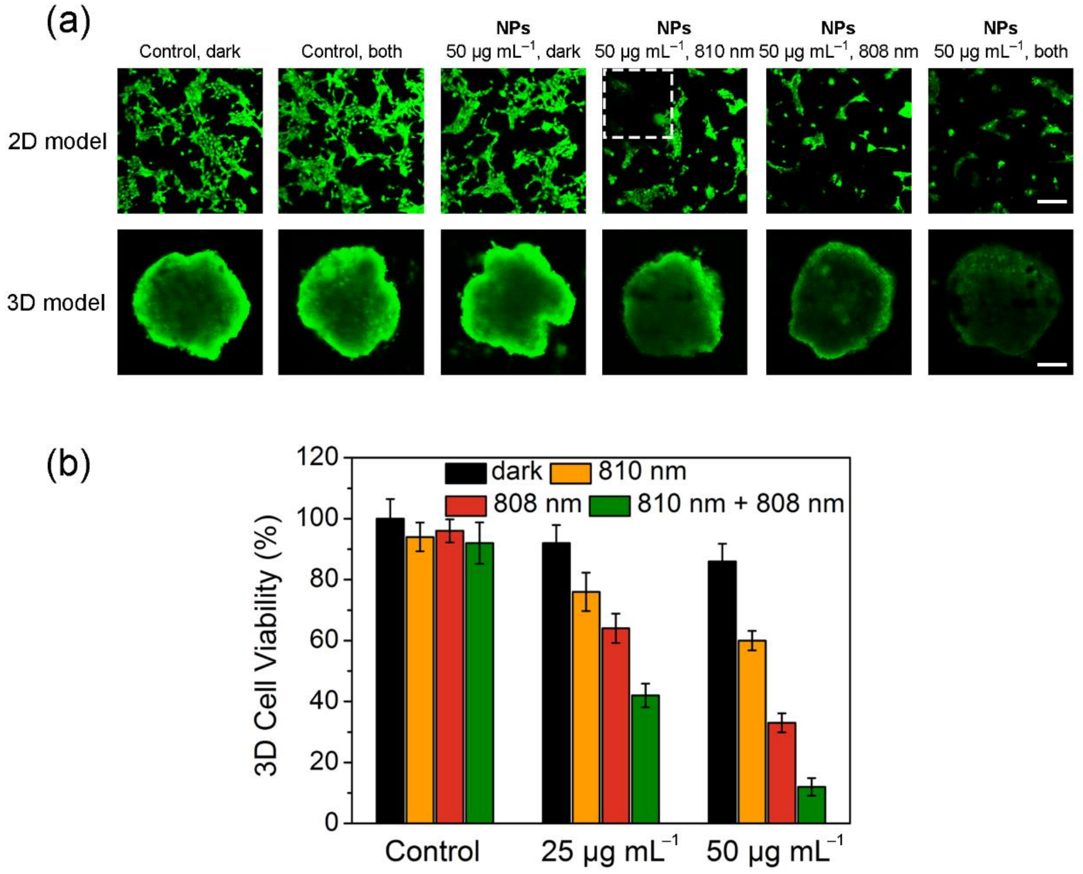

Figure S20. (a) Confocal microscopic images of the 4T1 cells and MCTSs stained with Calcein AM (λ_{ex} = 488 nm, λ_{em} = 520 ± 20 nm). The 2D cells and MCTSs were treated with **PDA-Pt-CD@RuFc** (50 μg mL^{-1}) for 24 h and then irradiated by the lasers. Irradiated conditions: 808 nm (1 W cm^{-2}, 10 min), 810 nm (100 mW, 80 MHz, 100 fs, 1 min (2D cells) or 20 min (MCTSs)). Scale bars: 100 μm. (b) Cell viability of MCTSs incubated with **PDA-Pt-CD@RuFc** NPs in the dark or upon light irradiation. The cells were irradiated with 808 nm laser (1 W cm^{-2}, 10 min) and a two-photon laser (810 nm, 100 mW, 80 MHz, 100 fs, 20 min) and further incubated for 24 h. Data are represented as mean ± SD (n = 3).

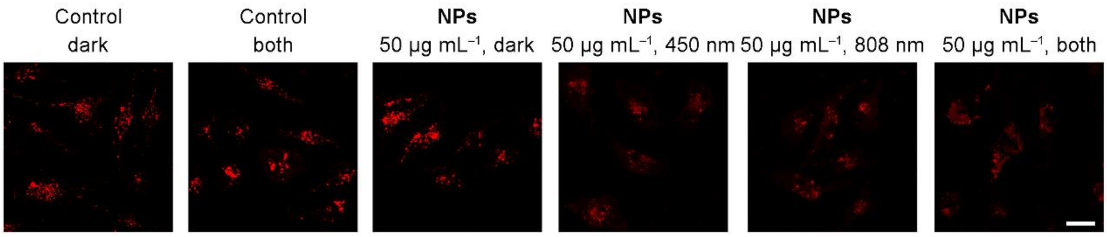

Figure S21. Observation of the release of cathepsin B from lysosomes to cytosol induced by **PDA-Pt-CD@RuFc**-mediated PDT-PTT in 4T1 cells using the fluorogenic substrate Magic Red MR-$(RR)_2$. Irradiation conditions: 450 nm, 17 mW cm^{-2}, 1 min. 808 nm, 1 W cm^{-2}, 10 min. Scale bar: 20 μm.

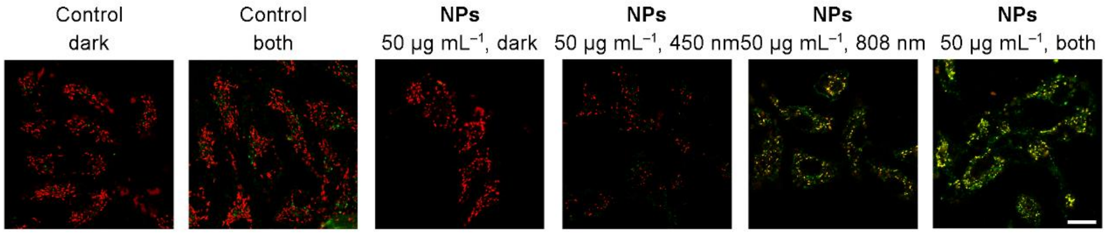

Figure S22. Detection of mitochondria membrane potential (MMP) in cells stained with JC-1 and analyzed by confocal microscopy after combined PDT-PTT treatment with **PDA-Pt-CD@RuFc** (50 μg mL^{-1}) in 4T1 cells. Irradiation conditions: 450 nm, 17 mW cm^{-2}, 1 min. 808 nm, 1 W cm^{-2}, 10 min. Scale bar: 20 μm.

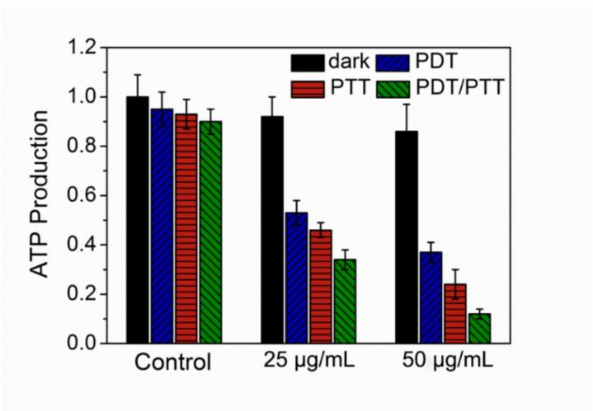

Figure S23. Intracellular ATP levels in 4T1 cells after PDT/PTT treatment with **PDA-Pt-CD@RuFc** NPs at the indicated concentrations. Irradiation conditions: 450 nm, 17 mW cm^{-2}, 1 min. 808 nm, 1 W cm^{-2}, 10 min.

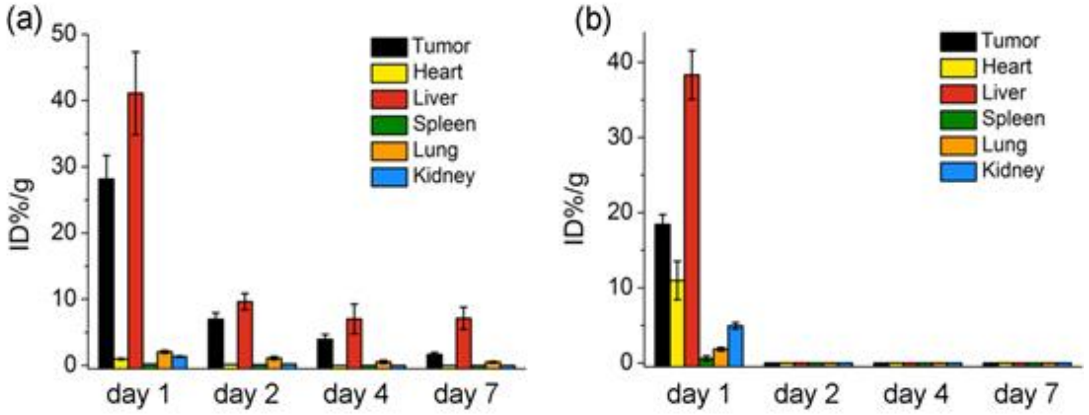

Figure S24. Biodistribution of (a) Pt and (b) Ru in different organs 7 days after i.v. injection of **PDA-Pt-CD@RuFc** NPs. The values were presented as the percentage of injected dose per g of the collected organs based on three mice per group.

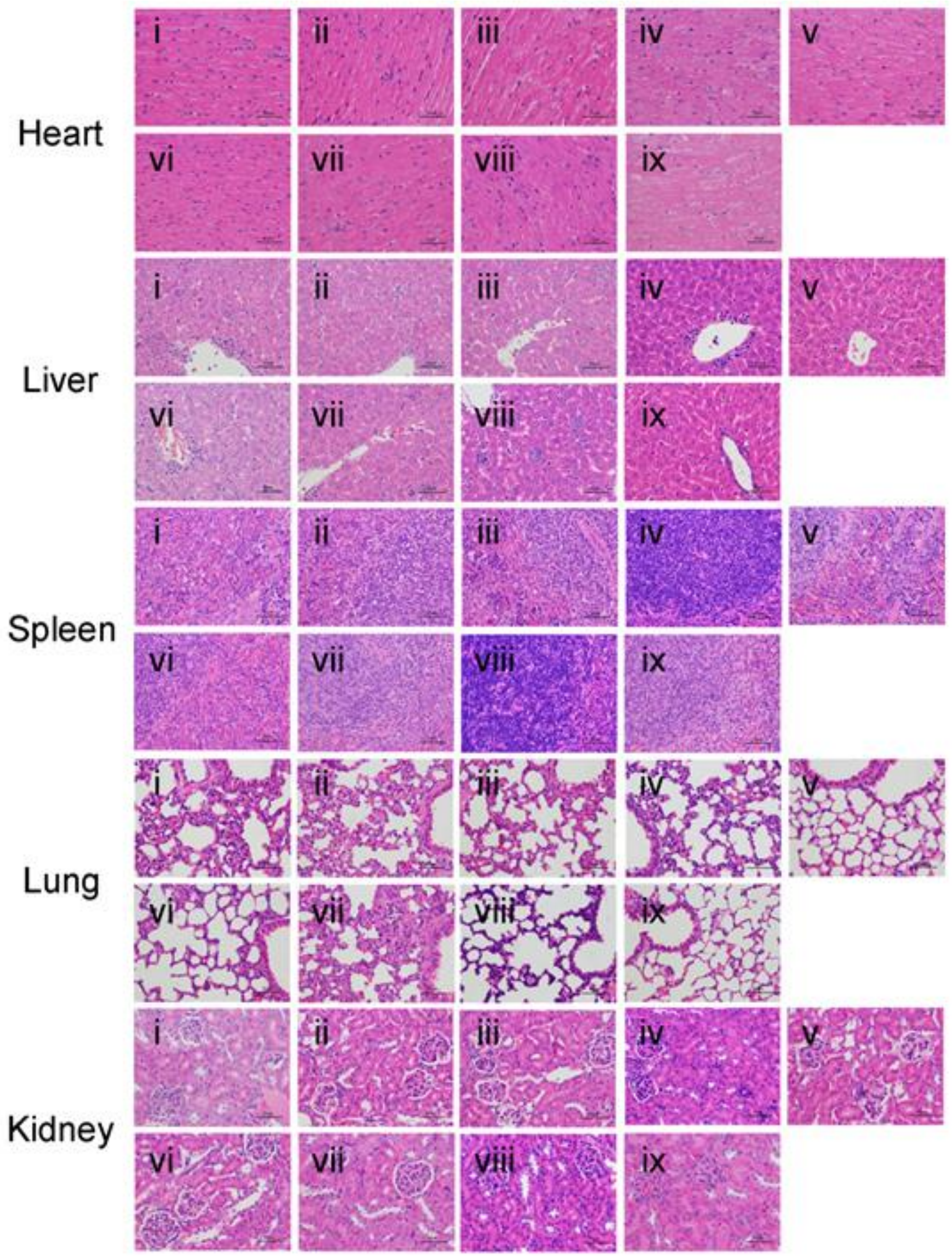

Figure S25. H&E stained slices of major organs of mice after combined PDT-PTT treatment mediated by **PDA-Pt-CD@RuFc** NPs. The mice are divided into nine groups: (i) control; **(**ii) dark, i.t.; (iii) 450 nm, i.t.; (iv) 808 nm, i.t.; (v) 808 nm + 450 nm, i.t.; (vi) dark, i.v.; (vii) 450 nm, i.v.; (viii) 808 nm, i.v.; (ix) 808 nm + 450 nm, i.v. Irradiation conditions: 450 nm, 12 W cm^{-2}, 5 min; 808 nm, 1 W cm^{-2}, 3 min. Scale bars: 50 µm.

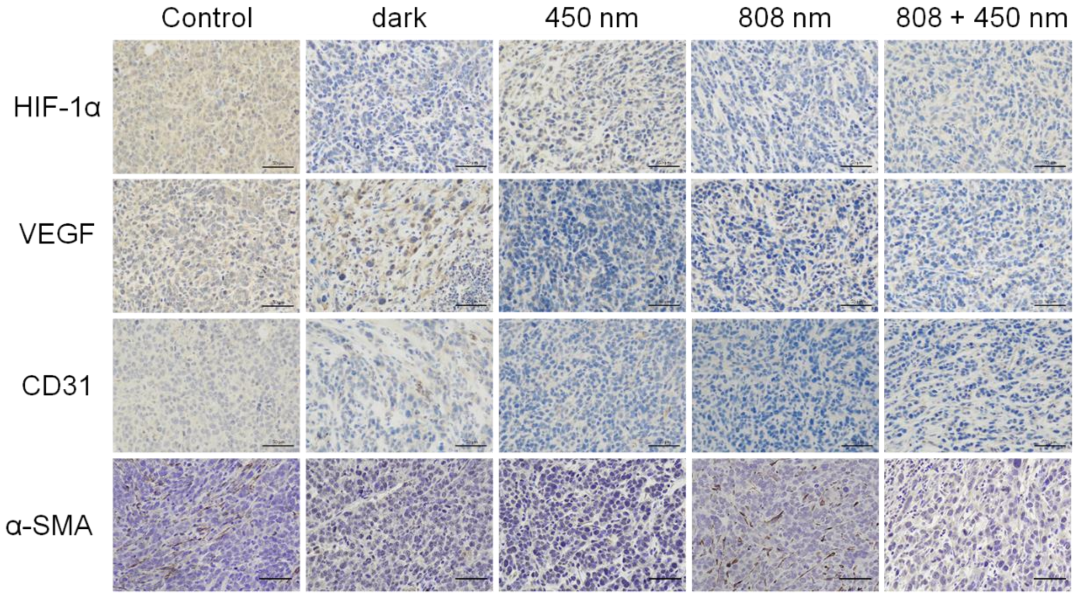

Figure S26. Immunohistochemical staining of CD31, HIF-1α, VEGF and α-SMA of 4T1 tumor specimens 24 h after the mice were intravenously injected with **PDA-Pt-CD@RuFc** NPs (200 μL, 1 mg mL^{-1}). Scale bars: 50 μm. Irradiation conditions: 450 nm, 12 W cm^{-2}, 5 min; 808 nm, 1 W cm^{-2}, 3 min.

Table S2. Photophysical data of **RuFc** in different solutions[a]

Compound	Medium	*τ (ns)*	Φ^{b}
**RuFc**	CH_2Cl_2	365.25	0.243
	CH_3CN	128.90	0.094
	PBS	35.21	0.022

[a]Measured in solvents degassed with prepurified argon gas. [b]The emission quantum yields at room temperature were determined using $[Ru(bpy)_3]Cl_2$ in aerated CH_2Cl_2 (Φ = 0.059), CH_3CN (Φ = 0.062) and H_2O(Φ = 0.028) [5] as the reference.

Table S3. $^1O_2$ quantum yields of **RuFc** in disodium hydrogen phosphate–citric acid buffer solutions

Complex	Medium	Φ_Δ[a]
**RuFc**	6.5	0.152
	7.4	0.098
**RuFc** + H_2O_2	6.5	0.226
	7.4	0.136

[a]The Φ_Δ were determined using $[Ru(bpy)_3]Cl_2$ in aerated H_2O (Φ_Δ = 0.18)[6] as the reference.

References

[1] D. Y. Zhang, Y. Zheng, C. P. Tan, J. H. Sun, W. Zhang, L. N. Ji, Z. W. Mao, *ACS Appl. Mater. Interfaces* **2017**, *9*, 6761.

[2] D. Y. Zhang, Y. Zheng, H. Zhang, L. He, C. P. Tan, J. H. Sun, W. Zhang, X. Y. Peng, Q. Q. Zhan, L. N. Ji, Z. W. Mao, *Nanoscale* **2017**, *9*, 18966.

[3] Y. Zheng, D. Y. Zhang, H. Zhang, J. J. Cao, C. P. Tan, L. N. Ji, Z. W. Mao, *Chem. Eur. J.* **2018**, *24*, 18971.

[4] D. Y. Zhang, Y. Zheng, H. Zhang, J. H. Sun, C. P. Tan, L. He, W. Zhang, L. N. Ji, Z. W. Mao, *Adv. Sci.* **2018**, *5*, 1800581.

[5] K. Nakamaru, *Bull. Chem. Soc. Jpn.* **1982**, *55*, 2697.

[6] J. M. Wessels, C. S. Foote, W. E. Ford, M. A. J. Rodgers, *Photochem. Photobiol.* **1997**, *65*, 96.
